# Closed-Loop Multiscale Computational Model of Human Blood Circulation. Applications to Ballistocardiography

**DOI:** 10.3389/fphys.2021.734311

**Published:** 2021-12-09

**Authors:** Jeremy Rabineau, Antoine Nonclercq, Tim Leiner, Philippe van de Borne, Pierre-Francois Migeotte, Benoit Haut

**Affiliations:** ^1^TIPs, Université Libre de Bruxelles, Brussels, Belgium; ^2^LPHYS, Université Libre de Bruxelles, Brussels, Belgium; ^3^BEAMS, Université Libre de Bruxelles, Brussels, Belgium; ^4^Department of Radiology, Utrecht University Medical Center, Utrecht, Netherlands; ^5^Department of Cardiology, Erasme Hospital, Université Libre de Bruxelles, Brussels, Belgium

**Keywords:** computational model, blood circulation, hemodynamics, aging, ballistocardiography, wearable cardiac monitoring, pulse wave velocity, stroke volume

## Abstract

Cardiac mechanical activity leads to periodic changes in the distribution of blood throughout the body, which causes micro-oscillations of the body’s center of mass and can be measured by ballistocardiography (BCG). However, many of the BCG findings are based on parameters whose origins are poorly understood. Here, we generate simulated multidimensional BCG signals based on a more exhaustive and accurate computational model of blood circulation than previous attempts. This model consists in a closed loop 0D-1D multiscale representation of the human blood circulation. The 0D elements include the cardiac chambers, cardiac valves, arterioles, capillaries, venules, and veins, while the 1D elements include 55 systemic and 57 pulmonary arteries. The simulated multidimensional BCG signal is computed based on the distribution of blood in the different compartments and their anatomical position given by whole-body magnetic resonance angiography on a healthy young subject. We use this model to analyze the elements affecting the BCG signal on its different axes, allowing a better interpretation of clinical records. We also evaluate the impact of filtering and healthy aging on the BCG signal. The results offer a better view of the physiological meaning of BCG, as compared to previous models considering mainly the contribution of the aorta and focusing on longitudinal acceleration BCG. The shape of experimental BCG signals can be reproduced, and their amplitudes are in the range of experimental records. The contributions of the cardiac chambers and the pulmonary circulation are non-negligible, especially on the lateral and transversal components of the velocity BCG signal. The shapes and amplitudes of the BCG waveforms are changing with age, and we propose a scaling law to estimate the pulse wave velocity based on the time intervals between the peaks of the acceleration BCG signal. We also suggest new formulas to estimate the stroke volume and its changes based on the BCG signal expressed in terms of acceleration and kinetic energy.

## Introduction

Computational models have become very attractive tools to complement scientific knowledge in parallel to clinical investigations at relatively low cost. Indeed, the research performed *in silico* often allows to evaluate the influence of different contributing factors to a given result. Hence, they help understanding the pathophysiological origin of some observations, as well as to predict the values or trends of some biomarkers in different hypothetical cases. One of the main advantages of computational models is that they allow to artificially set a condition that may be difficult to find in observational studies or non-ethical to implement in interventional studies. Once such models are validated for a given use, they provide a very efficient tool to quickly improve the understanding of the associated clinical situations.

Ballistocardiography (BCG) is an area of cardiology that would largely benefit from *in silico* investigations. BCG corresponds to the measurement of the periodical movements of the whole body in response to cardiovascular activity and especially the flow of blood in the vasculature (Inan et al., 2015). These movements with respect to the subject’s own body frame can be evaluated at the center of mass and can either be expressed as accelerations, velocities, displacements, forces, or energies. The first mention of these blood circulation-induced movements of the overall body was made at the end of the nineteenth century (Gordon, 1877). Then BCG received considerable attention in the middle of the following century, reaching its zenith with the extensive work of Starr and Noordergraaf measuring this signal on a suspended table (Starr and Noordergraaf, 1967). Brown and colleagues even defined different categories to distinguish normal from abnormal and potentially pathologic BCG tracings (Brown et al., 1950). It remains that this method was never adopted in clinical practice and faded away after the 1980s for several reasons: from the necessity to use cumbersome equipment, such as suspended beds, to the poor understanding of the physiological processes behind BCG signals, which prevented their proper interpretation, and the emergence of imagery techniques such as echocardiography (Giovangrandi et al., 2011). Nevertheless, recent technological developments have made possible much more accurate and easier ways to measure the BCG signal (Etemadi and Inan, 2018), which can now easily be recorded on multiple dimensions (Migeotte et al., 2016), including also rotations (Morra et al., 2020), while innovative metrics have been introduced with many potential applications, including telemedicine. New findings include, among others: the possibility to use BCG to optimize cardiac resynchronization therapy (Giovangrandi et al., 2011), a good correlation between some BCG parameters and the cardiac output (Inan et al., 2009a; Hossein et al., 2019), as well as the possibility to differentiate compensated from decompensated heart failure patients (Aydemir et al., 2020). Yet, many of these findings are based on parameters whose pathophysiological meaning is poorly understood. Inter-subject differences still need to be better understood, as well as the contribution of different physiological effects to the BCG signal. Indeed, as opposed to ECG, it is difficult to attribute a single physiological event to a wave on a BCG signal. Modern simulation tools could help bring a better understanding of the physiological origin of the BCG signal to offer this technology a diagnosis or even prognosis value.

Most of the attempts to mathematically simulate a BCG signal date back to the mid-twentieth century. Starr and Rawson first established a very basic model based on the contour of the cardiac ejection curve and including three elements: the heart, the aorta, and the pulmonary artery (Starr and Rawson, 1941). Since their results were relatively close to clinical observations, they concluded that these elements were the major contributors to the BCG signal and further detailed the respective contributions of such elements and their impact on the shape of this signal. After establishing the relationship between the compliance of arteries and their geometrical and elastic properties (Horeman and Noordergraaf, 1958), Noordergraaf and Horeman computed the evolution of blood volume in the large arteries and successfully compared their results with plethysmograms (Noordergraaf and Horeman, 1958). This served as a basis for computing the changes of blood mass occurring in 117 segments of the systemic and pulmonary circulations during a cardiac cycle (Noordergraaf et al., 1959). Using a fixed reference plane, they could then compute a simulated BCG signal in the longitudinal direction. Based on the equivalence between hydrodynamical and electrical systems, analog models have been developed, using resistors, inductors, and capacitors to model each arterial segment and account for the viscous and inertial properties of blood flow, as well as the elasticity of the arterial wall (Noordergraaf et al., 1963). Cardiac valves and peripheral vessels were simulated using diodes and resistors, respectively, while the contraction of cardiac chambers was modeled using time-varying capacitors (McLeod, 1964). The ability of such analog models to generate a simulated BCG signal on the longitudinal direction was successfully tested using actual electrical circuits (Starr and Noordergraaf, 1967) and early numerical simulations (Auslander et al., 1972). However, these simulations were all based on approximate anatomical data and computed in one representative reference case.

Since then, computational models of blood circulation have improved a lot and found numerous applications (Liang et al., 2009a; van de Vosse and Stergiopulos, 2011; Scarsoglio et al., 2018; Charlton et al., 2019). However, these models were often limited to the computation of blood flow and pressure at various locations of the circulatory system, without any immediate possibility to apply these results to BCG. A mathematical model limited to the aorta has recently been able to reproduce the classical shape of the BCG signal, even though some waves were absent (Kim et al., 2016). Such a model allows for basic interpretation of the BCG signal, even though it is based on the assumption that the BCG signal is only generated by pressure gradients in the aorta. Moreover, it relies on the prior knowledge of pressure waves measured intrusively at different locations (Yousefian et al., 2019). The results from a more complex closed-loop system considering the contribution to the BCG signal from the left and right ventricles, the pulmonary trunk, as well as six arteries of the systemic circulation, have been published by Guidoboni et al. (2017). Still, the latter model includes several simplifications, such as the assumption that only a small selection of blood compartments has an influence on the BCG signal. Besides this, most of the models so far have focused only on the head-to-foot component of the acceleration BCG signal, while modern devices can measure BCG in up to three linear and three rotational dimensions (Migeotte et al., 2016; Hossein et al., 2019), showing high repeatability (Hossein et al., 2021a).

Based on this literature review, the general objective of this work is to propose a computational model of blood flow in the circulatory system and to use this model to give further insights into the physiological origin of the multidimensional BCG signal. Consequently, our first goal is to provide a new mathematical model allowing a comprehensive analysis of the different elements affecting the BCG signal, with a strong physiological and anatomical background, which is useful for the qualitative and quantitative analysis of such signals. Our second objective is then to use this model to highlight the key parameters governing the generation of the BCG signal. Finally, our third objective is to use the model to provide new elements to understand the impact of age on the BCG signal, as well as the relationship between some classical cardiological parameters and BCG-based metrics.

## Materials and Methods

### General Overview of the Model

The cardiac mechanical activity leads to periodic changes in the distribution of blood through the body, in particular in the cardiac chambers, the thoracic and abdominal arteries, and the extremities (Starr and Noordergraaf, 1967). These circulatory events result in oscillations of the body’s center of mass during each cardiac cycle, which can be evaluated by BCG. The position of this center of mass can also be periodically modified by other phenomena, such as respiratory movements or displacement of the heart and the vessels themselves in reaction to the cardiac and respiratory activities (Starr and Noordergraaf, 1967). However, we neglect these effects in the model. Since the considered periods of time are sufficiently short for the total body mass to stay constant, we assume that the BCG signal depends solely on blood movements in the cardiac chambers and in the vessels exposed to significantly pulsatile flows, i.e., the arteries.

**FIGURE 1:**
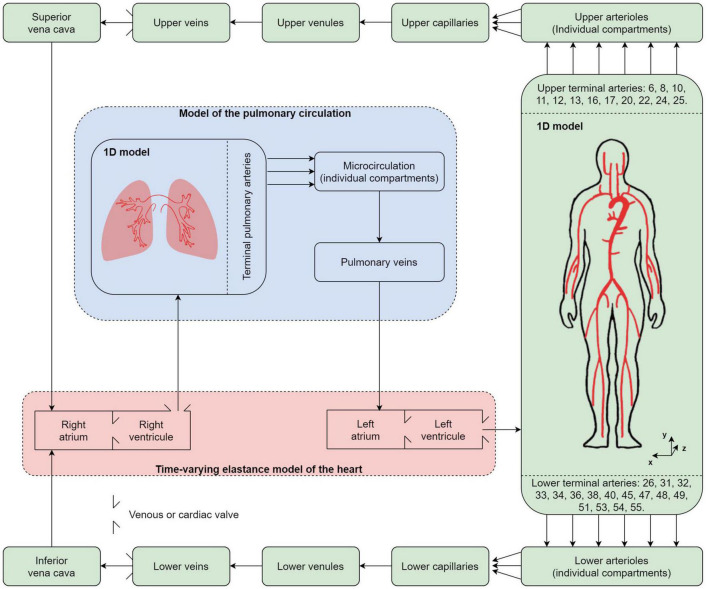
Different compartments used in the multidimensional computational model of blood circulation. Green background: systemic circulation; blue background: pulmonary circulation; red background: cardiac circulation including four cardiac valves. 55 systemic and 57 pulmonary arteries are considered in the 1D models of the systemic and pulmonary arterial circulation, respectively.

The Figure 1 describes the proposed computational model of blood circulation. It is based on a closed loop 0D-1D multiscale representation. The 55 main arteries of the systemic circulation and the 57 largest arteries of the pulmonary circulation are considered as 1D elements. The 0D elements include the individual arteriolar compartments after each terminal artery, as well as the capillaries, venules, veins, and cardiac chambers and their valves. As opposed to open-loop models restricted on the systemic arterial tree or ventricular-arterial models, such a closed-loop representation does not require boundary conditions. This solves the issue of potential mismatch between fixed boundary conditions and changes in the input parameters of the model (heart rate, pulse wave velocities, etc.).

The equations of blood flow (see Sections “Model of the Heart” and “Model of the Vasculature”) are solved to compute the distribution of blood through a cardiac cycle in the different compartments. Then, these signals are associated to the tridimensional anatomical position of the different compartments, which allows the computation of a 3D BCG signal (see Section “Simulated Ballistocardiography Signal”). As of now, and in order to better separate the contribution of different parameters on the output signal, regulatory mechanisms such as the baroreflex are not included in the model, and only periodic solutions are considered. By periodic, we mean a state of the system such that a cardiac cycle is sufficiently similar to the previous one, which is systematically the case after less than 10 s of simulated time.

### Model of the Heart

#### Cardiac Chambers

A basic time-varying elastance model is chosen for each of the four cardiac chambers (Suga et al., 1973). In this model, the pressure inside a given cardiac chamber (*P*_*CC*_) is described by the following relationship, neglecting any viscoelastic effect:


(1) $$P_{C\,C}\,\left(t\right)=E_{C\,C}\,\left(t\right)\,\left[V_{C\,C}\,\left(t\right)-V_{\text{dead},C\,C}\right]$$

where *t* is the time, *V*_*CC*_ the instantaneous volume of the cardiac chamber, *V*_*dead, CC*_ its dead volume (or zero-pressure volume), and *E_CC_* its time-varying elastance, given by Liang et al. (2009a):


(2) $$E_{C\,C}\,\left(t\right)=E_{A,C\,C}\,e_{C\,C}\,\left(t\right)+E_{B,C\,C}$$

where *E*_*A, CC*_ is the amplitude of the variation of elastance associated to the cardiac chamber, *E*_*B, CC*_ its baseline value, and *e*_*CC*_ is a normalized time-varying function. *e*_*CC*_(*t*) is defined as in Liang et al. (2009a) (see Section 2 of the Supplementary Material). We consider that the left and right ventricles (CC = LV and RV, respectively) contract simultaneously, at the very beginning of the cardiac cycle (of length *T*_*RR*_). This contraction period of length *T*_*vcp*_ is then immediately followed by a relaxation period of length *T*_*vrp*_. The assumptions are similar for the left and right atria (CC = LA and RA, respectively), with a contraction period of length *T*_*acp*_ starting at 0.8*T*_*RR*_ (Gallo et al., 2020), immediately followed by a relaxation period of length *T*_*arp*_. The numerical values used to model the time-varying elastance in each cardiac chamber are taken from Liang et al. (2009a) and given in Table 1 for the case of normal contractility and *T*_*RR*_ = 0.86 s (corresponding to a heart rate of 70 bpm). An increase of contractility results in shorter contraction and relaxation phases and larger amplitudes of elastance (Suga et al., 1973). An increase in heart rate results only in shorter contraction and relaxation phases (Suga et al., 1973). The actual impact of the heart rate on the duration of the contraction and relaxation periods is chosen as described by Gallo and coworkers: $T_{v\,c\,p}=0.3\,\sqrt{T_{R\,R}}$; *T*_*vrp*_ = 0.5 *T*_*vcp*_; *T*_*acp*_ = 0.17*T*_*RR*_; and *T*_*arp*_ = *T*_*acp*_ (Gallo et al., 2020).

**TABLE 1:** Numerical values of the parameters used in the model of the heart for a cardiac cycle of duration *T*_*RR*_ = 0.86 s (corresponding to a heart rate of 70 bpm) in a normal contractility context.

	Right atrium	Right ventricle	Left atrium	Left ventricle	Tricuspid valve	Pulmonary valve	Mitral valve	Aortic valve
*V*_*dead*_ (ml)	7.0	40.0	3.0	10.0	−	−	−	−
*E*_*A, CC*_ (mmHg.ml^–1^)	0.06	0.55	0.07	2.75	−	−	−	−
*E*_*B, CC*_ (mmHg.ml^–1^)	0.07	0.05	0.09	0.10	−	−	−	−
*T*_*acp*_, *T*_*vcp*_ (s)	0.15	0.28	0.15	0.28	−	−	−	−
*T*_*arp*_, *T*_*vrp*_ (s)	0.15	0.14	0.15	0.14	−	−	−	−
*A*_eff,max,*CV*_(cm^2^)	−	−	−	−	6.0	5.7	5.1	5.0
*l*_*eff, CV*_ (cm)	−	−	−	−	2.0	1.5	2.0	1.0
*K*_*vo, CV*_ (mmHg^–1^.s^–1^)	−	−	−	−	40.0	26.7	26.7	26.7
*K*_*vc, CV*_ (mmHg^–1^.s^–1^)	−	−	−	−	53.3	26.7	53.3	26.7

***E_A,CC_**: amplitude of elasticity; **E_B,CC_**: baseline value of elasticity; **T_vcp_**, **T_acp_**: duration of the contraction phase for the ventricles and atria, respectively; **T_vrp_**, **T_arp_**: duration of the relaxation phase for the ventricles and atria, respectively; **A_eff,max,CV_**: maximal effective area; **l_eff,CV_**: effective length; **K_vo,CV_**: rate coefficient for valve opening; **K_vc,CV_**: rate coefficient for valve closing. Numerical values for the cardiac valves and dead volumes are adapted from Mynard and Smolich (2015), while all the other numerical values are adapted from Liang et al. (2009a).*

In this model, pressure coupling through the interventricular septum and volume coupling inside the pericardium are assumed to have a negligible effect on intracardiac hemodynamics. The baroreflex control of the heart rate and the effect of intrathoracic pressure (modulated via breathing) are not considered. For more information regarding the modeling of these additional effects, see (Sun et al., 1997).

#### Cardiac Valves

In the nominal case of a healthy heart, the pressure drop through a cardiac valve △*P*_*CV*_ is expressed as in Mynard et al. (2012), keeping their assumption that Poiseuille-type viscous losses can be neglected:


(3) $$△\,P_{C\,V}\,\left(t\right)=B_{C\,V}\,\left(t\right)\,Q_{C\,V}\,\left(t\right)\,\left|Q_{C\,V}\,\left(t\right)\right|+L_{C\,V}\,\left(t\right)\,{\dot{Q}}_{C\,V}\,\left(t\right)$$

where *B*_*CV*_ is the coefficient of the flow separation (or Bernoulli) term, *L*_*CV*_ the coefficient of the inertial term, and *Q*_*CV*_ the blood flow rate through the cardiac valve (positive when blood is flowing in the physiological direction).

*B*_*CV*_ is given by the following relationship:


(4) $$B_{C\,V}\,\left(t\right)=\frac{\rho }{2\,A_{\mathrm{eff},C\,V}\,{\left(t\right)}^{2}}$$

with *A*_*eff*,*CV*_(*t*) a time-varying effective cross-sectional area for the cardiac valve and ρ=1050*kg*.^m−3^ the density of blood at 37°C (Pedley, 1980).

*L*_*CV*_ is given by the following relationship:


(5) $$L_{C\,V}\,\left(t\right)=\frac{\rho \,l_{\text{eff},C\,V}}{A_{\text{eff},C\,V}\,\left(t\right)}$$

with *l*_*eff, CV*_ a constant effective length for the cardiac valve.

The dynamics of the cardiac valves is also considered to follow the representation suggested in Mynard et al. (2012), with the additional assumption that the four cardiac valves are always able to open and close entirely. By defining a valve state η_*CV*_ with 0 ≤ η _*CV*_ ≤   1, η _*CV*_ = 0 when the valve is totally closed, and η_*CV*_ = 1 when it is totally open, the effective cross-sectional area is given by:


(6) $$A_{\mathrm{eff},C\,V}\,\left(t\right)=\eta_{C\,V}\,\left(t\right)\,A_{\text{eff},\max,C\,V}$$

with *A*_*eff,max, CV*_ the maximal effective area of the cardiac valve. The numerical values of both *A*_*eff,max, CV*_ and *l*_*eff, CV*_ are given in Table 1 for each cardiac valve.

The cardiac valves are assumed to open as soon as △*P*_*CV*_ exceeds a positive threshold △*P*_open,*CV*_, and to close as soon as △*P*_*CV*_ is lower than a negative threshold △*P*_close,*CV*_. The following relationship describes this behavior:


(7) $$\frac{d\,\eta_{C\,V}\,\left(t\right)}{d\,t}=\left\{ \begin{array}{c} \left(1-\eta_{C\,V}\,\left(t\right)\right)\,K_{v\,o,C\,V}\,\left(△\,P_{C\,V}\,\left(t\right)-△\,P_{\text{open},C\,V}\right)\\ w\,h\,e\,n\\ △\,P_{C\,V}>△\,P_{\text{open},C\,V}\\ 0\\ w\,h\,e\,n\\ △\,P_{\text{close},C\,V}\leq △\,P_{C\,V}\leq △\,P_{\text{open},C\,V}\\ \eta_{C\,V}\,\left(t\right)\,K_{v\,c,C\,V}\,\left(△\,P_{C\,V}\,\left(t\right)-△\,P_{\text{close},C\,V}\right)\\ w\,h\,e\,n\\ △\,P_{C\,V}<△\,P_{\text{close},C\,V} \end{array}\right.$$

with *K*_*vo, CV*_ the rate coefficient for valve opening, *K*_*vc, CV*_ the one for valve closing (see their values in Table 1). We also assume that △*P*_close,*CV*_ = △*P*_open,*CV*_ = 0 for all the cardiac valves.

### Model of the Vasculature

#### 1D Model of Blood Flow Through a Single Artery

**FIGURE 2:**
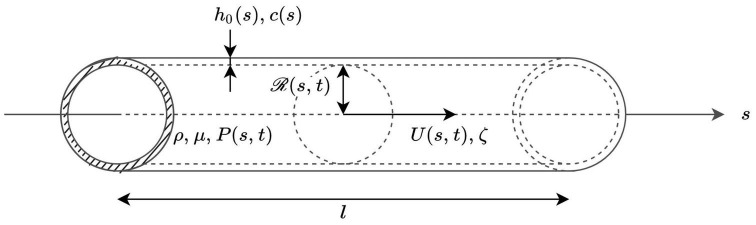
Local representation of blood flow in an artery, whose properties are defined by the axial coordinate *s*. ρ and μ are the volumetric mass density and dynamic viscosity of blood, respectively. At each cross-section, the wall thickness *h*_0_(*s*) as well as the pulse wave velocity *c*(*s*) are time independent. The lumen radius at position *s* and time *t* is noted 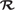
(*s*,*t*), while the cross-sectional average of the velocity and of the blood pressure at position *s* and time *t* are noted *U*(*s*,*t*) and *P*(*s*,*t*), respectively. ζ is a parameter that is constant for each artery and defines the velocity profile.

Each of the considered arteries is modeled as an axisymmetric tube of constant length *l* oriented along a *s* axis pointing in the distal direction, with a lumen radius that varies with position *s* and time *t* (see Figure 2). An axisymmetric distribution of all the parameters is assumed. The axial (*s*) component of the blood velocity in the artery is assumed to be predominant in comparison with the radial (*r*) component (which is thus neglected), while vessel wall movements because of changes in internal blood pressure are considered to occur only in the radial direction (Smith et al., 2002).

For a given artery, the radius of the lumen, the cross-sectional average of the blood velocity, and the blood pressure at position *s* and time *t* are written ℛ(*s*,*t*), *U*(*s*,*t*), and *P*(*s*,*t*), respectively. The cross-sectional area of the lumen is noted *A*(*s*,*t*) = π ℛ(*s*,*t*)^2^ and the blood flow rate at position *s* and time *t* is written *Q*(*s*,*t*), with *Q*(*s*,*t*) = *U*(*s*,*t*) *A*(*s*,*t*).

Under the assumptions that blood is an homogeneous, incompressible, and Newtonian fluid in medium and large arteries (Caro et al., 2012), that the effect of gravity is negligible (supine position), and that there is no seepage of blood through the vessel walls, mass and momentum balances give the following equations to describe the flow in the artery (Sherwin et al., 2003):


$$\{\begin{array}{cc} \frac{\partial A(s,t)}{\partial t}+\frac{\partial Q(s,t)}{\partial s}=0 & \left(8\right)\\ \frac{\partial Q(s,t)}{\partial t}+\frac{\partial }{\partial s}(\text{α}\frac{Q{\left(s,t\right)}^{2}}{A(s,t)})+\frac{A(s,t)}{\text{ρ}}\frac{\partial P(s,t)}{\partial s}=\frac{f(s,t)}{\text{ρ}} & \left(9\right) \end{array}$$

where *f*(*s*,*t*) is the friction force per unit length of the blood flow on the arterial wall, and α is the Coriolis coefficient. To determine these two parameters, a given shape of the velocity profile in the artery must be assumed. Similarly to previous studies (Hughes and Lubliner, 1973), the following expression of this velocity profile, with a no-slip condition at the wall, is used:


(10) $$u\,\left(s,r,t\right)=\frac{\zeta +2}{\zeta }\,\left(1-{\left(\frac{r}{ℛ\,\left(s,t\right)}\right)}^{\zeta }\right)\,U\,\left(s,t\right)$$

with *u*(*s*,*r*,*t*) the axial component of the blood velocity at axial position *s*, radial position *r*, and time *t*, while ζ is a constant that can be related to the Coriolis coefficient. Indeed, if such an expression of the velocity profile is used, we can write $\alpha =\frac{\zeta +2}{\zeta +1}$ (Alastruey et al., 2012).

Consequently, the friction force per unit length at the wall can be written as (Smith et al., 2002):


(11) $$f\,\left(s,t\right)={2\,\pi \,ℛ\,\left(s,t\right)\,\mu \,\frac{\partial u}{\partial r}|}_{r=ℛ}=-2\,\left(\zeta +2\right)\,\mu \,\pi \,U\,\left(s,t\right)$$

where μ = 4.0 × 10^−3^ *kg*.m^−1^ .s^−1^ is the dynamic viscosity of blood at 37°C (Pedley, 1980), assumed to be constant (Quarteroni et al., 2000).

In the case of a fully developed Poiseuille flow, ζ=2 (i.e., α=4/3). However, depending on the hemodynamic conditions and the vessel size, the velocity profile does not necessarily correspond to a Poiseuille flow. It has been shown that a good fit to experimental data is obtained with ζ = 9 (i.e., α = 1.1) (Smith et al., 2002) but, actually, the value of ζ should be adapted to the flow conditions in each artery, as they might differ a lot between the aorta and the smaller arteries. Consequently, in this model, we first solve the equations considering a fully established Poiseuille flow in each of the arteries (i.e., ζ = 2 for all the arteries). Then, in a second step and for each artery *i*, we compute the minimal length *l*_*P, i*_ necessary to establish a Poiseuille flow, using the following equation (see Section 4 in the Supplementary Material):


(12) $$l_{P,i}=\frac{\rho \,{ℛ}_{\text{prox},i}^{2}\,{\bar{U}}_{\text{prox},i}}{4\,\mu }$$

where ${\bar{U}}_{\text{prox},i}$ is the time average of *U* taken at the proximal end of the artery *i* and ℛ_prox,*i*_ is the radius of the artery *i* at its proximal end and at a reference pressure *P*_*ref*_ (usually the diastolic pressure).

Then, and only if the length of the artery *l_i_* is smaller than *l*_*P, i*_, we update, as described in the Supplementary Material, the value of the coefficient ζ for the artery *i* as follows, based on a method described in the Supplementary Material of Buess et al. (2020):


(13) $$\zeta_{i}=4\,\sqrt{\frac{l_{P,i}}{l_{i}}}-2$$

Then the equations of the model are solved once again with the updated values of ζ_*i*_.

In agreement with *in vivo* observation, the calculations give a relatively blunt profile (i.e., large values of ζ) in the large proximal systemic arteries such as the aorta (Seed and Wood, 1971; Bogren and Buonocore, 1999; Tortoli et al., 2002) and a parabolic profile (i.e., ζ = 2) in more distal arteries (Nichols et al., 2011).

We also define a Voigt-type visco-elastic constitutive law between *P*(*s*,*t*) and *A*(*s*,*t*), and neglect the influence of surrounding tissues. Assuming a thin, isotropic, homogeneous, and incompressible vessel wall, where each section is independent of the others and has constant structural properties, we can write that (Alastruey et al., 2012):


(14) $$\begin{array}{l} P(s,t)=P_{ref}+\frac{\text{β}(s)}{A_{0}(s)}(\sqrt{A(s,t)}-\sqrt{A_{0}(s)})\\ \;+\frac{\text{Γ}(s)}{A_{0}(s)\sqrt{A(s,t)}}\frac{\partial A(s,t)}{\partial t} \end{array}$$

where *A*_0_(*s*) is the cross-sectional area when *P*(*s*,*t*) = *P*_ref_ and $\frac{\partial A\,\left(s,t\right)}{\partial t}=0$, β(*s*) is a parameter related to the arterial wall elasticity, and Γ(*s*) is a parameter related to the arterial wall viscosity. We can also introduce $P_{\text{elast}}=\frac{\beta \,\left(s\right)}{A_{0}\,\left(s\right)}\,\left(\sqrt{A\,\left(s,t\right)}-\sqrt{A_{0}\,\left(s\right)}\right)$.

Based on the pressure-area relationship, Equations 8, 9 can then be linearized locally around the diastolic state and give (Alastruey et al., 2012):


$$\{\begin{array}{cc} \frac{2A_{0}{\left(s\right)}^{3/2}}{\text{β}(s)}\frac{\partial P_{elast}(s,t)}{\partial t}+\frac{\partial Q(s,t)}{\partial s}=0 & \left(15\right)\\ \frac{\rho }{A_{0}(s)}\frac{\partial Q(s,t)}{\partial t}+\frac{\partial P_{elast}(s,t)}{\partial s}-\frac{\text{Γ}(s)}{A_{0}{\left(s\right)}^{3/2}}\frac{\partial^{2}Q(s,t)}{\partial s^{2}}=-\frac{2(\text{ζ}+2)\text{πμ}}{A_{0}{\left(s\right)}^{2}}Q(s,t) & \left(16\right) \end{array}$$

The method used to obtain *A*_0_(*s*), β(*s*), and Γ(*s*) in each artery is described in Section “Anatomical and Physiological References” and is mostly based on magnetic resonance (MR) angiography data. The supporting numerical values are given in Table 2 for the systemic arteries and Table 3 for the pulmonary arteries.

**TABLE 2:** Anatomical and physiological data used in the 1D model of the systemic arterial tree, based on magnetic resonance angiography on a healthy female (25 years old, 172 cm, 71 kg).

No	Systemic artery	Mother artery	*l* (cm)	ℛ_prox_→ℛ_dist_ (cm)	*R*_0_, *R_1_* (mmHg.s.ml^–1^)	*C*_1_ (ml.mmHg^–1^)
1	Ascending aorta	−	4.4	1.092 → 1.193	−	−
2	Aortic arch I	1	1.0	1.157 → 1.072	−	−
3	Brachiocephalic	1	3.7	0.881 → 0.296	−	−
4	R. subclavian I	3	2.9	0.405 → 0.438	−	−
5	R. carotid	3	10.3	0.312 → 0.327	−	−
6	R. vertebral	4	23.4	0.249 → 0.148	6.10, 27.87	0.0126
7	R. subclavian II^*^	4	39.8	0.410 → 0.229	−	−
8	R. radial^†^	7	22.0	0.175 → 0.140	14.21, 18.34	0.0143
9	R. ulnar I^†^	7	6.7	0.215 → 0.215	−	−
10	R. interosseous^†^	9	7.0	0.100 → 0.100	39.43, 424.01	0.0009
11	R. ulnar II^†^	9	17.0	0.203 → 0.180	10.12, 21.20	0.0143
12	R. internal carotid	5	15.8	0.268 → 0.162	2.53, 25.42	0.0148
13	R. external carotid	5	12.0	0.204 → 0.109	5.23, 23.53	0.0148
14	Aortic arch II	2	1.5	1.096 → 1.046	−	−
15	L. carotid	2	13.4	0.394 → 0.262	−	−
16	L. internal carotid	15	15.1	0.262 → 0.175	2.53, 25.42	0.0148
17	L. external carotid	15	11.4	0.177 → 0.103	5.23, 23.53	0.0148
18	Thoracic aorta I	14	7.9	1.078 → 0.850	−	−
19	L. subclavian I	14	5.2	0.731 → 0.238	−	−
20	L. vertebral	19	20.8	0.173 → 0.164	6.10, 27.87	0.0126
21	L. subclavian II^*^	19	39.8	0.410 → 0.228	−	−
22	L. radial^†^	21	22.0	0.175 → 0.140	14.21, 18.34	0.0143
23	L. ulnar I^†^	21	6.7	0.215 → 0.215	−	−
24	L. interosseous^†^	23	7.0	0.100 → 0.100	39.43, 424.01	0.0009
25	L. ulnar II^†^	23	17.0	0.203 → 0.180	10.12, 21.20	0.0143
26	Intercostals^†^	18	7.3	0.300 → 0.300	2.00, 6.04	0.0542
27	Thoracic aorta II	18	15.9	0.909 → 0.805	−	−
28	Abdominal aorta I	27	1.3	0.815 → 0.790	−	−
29	Celiac I	27	1.8	0.614 → 0.233	−	−
30	Celiac II^†^	29	2.0	0.300 → 0.250	−	−
31	Hepatic^†^	29	6.5	0.275 → 0.250	2.80, 17.48	0.0208
32	Gastric^†^	30	5.5	0.200 → 0.200	4.05, 9.59	0.0325
33	Splenic^†^	30	5.8	0.175 → 0.150	8.59, 22.97	0.0139
34	Superior mesenteric	28	12.5	0.413 → 0.115	1.20, 4.15	0.0810
35	Abdominal aorta II	28	0.5	0.795 → 0.788	−	−
36	L. renal	35	5.9	0.458 → 0.161	2.02, 4.64	0.0667
37	Abdominal aorta III	35	1.5	0.769 → 0.734	−	−
38	R. renal	37	5.0	0.424 → 0.215	2.02, 4.64	0.0667
39	Abdominal aorta IV	37	7.0	0.735 → 0.696	−	−
40	Inferior mesenteric	39	3.7	0.343 → 0.036	5.37, 33.10	0.0110
41	Abdominal aorta V	39	2.5	0.699 → 0.582	−	−
42	L. common iliac	41	7.6	0.461 → 0.374	−	−
43	R. common iliac	41	6.9	0.416 → 0.395	−	−
44	L. external iliac	42	13.3	0.335 → 0.391	−	−
45	L. internal iliac	42	8.1	0.316 → 0.058	6.40, 25.28	0.0136
46	L. femoral	44	45.0	0.312 → 0.256	−	−
47	L. deep femoral	44	20.0	0.373 → 0.160	4.99, 14.40	0.0226
48	L. posterior tibial	46	35.9	0.202 → 0.117	9.90, 32.78	0.0102
49	L. anterior tibial	46	35.9	0.139 → 0.109	3.96, 15.11	0.0226
50	R. external iliac	43	16.2	0.321 → 0.451	−	−
51	R. internal iliac	43	9.8	0.284 → 0.123	6.40, 25.28	0.0136
52	R. femoral	50	49.3	0.327 → 0.250	−	−
53	R. deep femoral	50	21.6	0.482 → 0.160	4.99, 14.40	0.0226
54	R. posterior tibial	52	27.6	0.189 → 0.117	9.90, 32.78	0.0102
55	R. anterior tibial	52	27.8	0.157 → 0.112	3.96, 15.11	0.0226

*The reference pressure is assumed to be 70 mmHg. Complete dataset of the segmented arteries, including the evolution of radius, 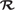
(s), and the position of the centerline, **G**(s), along each segmented artery, is available as Supplementary Material. **l**: length of the artery; 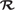
_prox_: radius of the lumen at the proximal end; 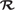
_dist_: radius of the lumen at the distal end; **R_0_**, **R_1_**: resistances in the arteriolar compartments following terminal arteries (see Equation 19); **C_1_**: peripheral compliance associated to the arterioles. Peripheral parameters are taken from Liang et al. (2009a).*

^†^Artery considered in the 1D model, but not segmented and thus not included in the BCG computation. *Artery partially segmented and extrapolated to be included in the BCG computation. Geometric data for non- or partially segmented arteries are taken from Liang et al. (2009a).

**TABLE 3:** Anatomical and physiological data used in the 1D model of the pulmonary arterial tree.

No	Pulmonary artery	Mother artery	*l* (cm)	ℛ (cm)	*R*_0_, *R*_1_ (mmHg.s.ml^–1^)	*C*_1_ (ml.mmHg^–1^)
1	Main pulmonary	−	4.90	1.35	−	−
2	L. pulmonary	1	2.60	0.90	−	−
3	R. pulmonary	1	3.09	1.10	−	−
4	L. inferior pulmonary	2	1.70	0.84	−	−
5	L. superior pulmonary	2	0.80	0.48	−	−
6	R. inferior pulmonary	3	2.41	0.92	−	−
7	R. superior pulmonary	3	2.00	0.76	−	−
8	LIA1_0_2^†^	4	1.93	0.76	−	−
9	LIA0_1_3^†^	4	1.31	0.51	−	−
10	LIA2_0_4^†^	8	1.74	0.68	−	−
11	LIA1_1_5^†^	8	1.18	0.46	0.39, 1.3	0.69
12	LIA3_0_6^†^	10	1.56	0.61	−	−
13	LIA2_1_7^†^	10	1.06	0.42	0.51, 1.6	0.56
14	LIA4_0_8^†^	12	1.40	0.55	−	−
15	LIA3_1_9^†^	12	0.95	0.37	0.67, 2.0	0.45
16	LIA5_0_10^†^	14	1.26	0.49	0.33, 1.1	0.79
17	LIA4_1_11^†^	14	0.86	0.34	0.88, 2.5	0.37
18	LIA1_1_12^†^	9	1.18	0.46	0.39, 1.3	0.69
19	LIA0_2_13^†^	9	0.79	0.31	1.05, 2.9	0.32
20	LSA1_0_15^†^	5	1.10	0.43	−	−
21	LSA0_1_16^†^	5	0.75	0.29	1.25, 3.3	0.72
22	LSA2_0_17^†^	20	0.99	0.39	−	−
23	LSA1_1_18^†^	20	0.67	0.26	1.64, 4.0	0.58
24	LSA3_0_19^†^	22	0.89	0.35	−	−
25	LSA2_1_20^†^	22	0.61	0.24	2.16, 5.0	0.47
26	LSA4_0_21^†^	24	0.80	0.31	1.04, 2.8	0.83
27	LSA3_1_22^†^	24	0.54	0.21	2.83, 6.2	0.38
28	RIA1_0_24^†^	6	2.11	0.83	−	−
29	RIA0_1_25^†^	6	1.43	0.56	−	−
30	RIA2_0_26^†^	28	1.90	0.75	−	−
31	RIA1_1_27^†^	28	1.29	0.51	−	−
32	RIA3_0_28^†^	30	1.71	0.67	−	−
33	RIA2_1_29^†^	30	1.16	0.45	0.41, 1.4	0.86
34	RIA4_0_30^†^	32	1.54	0.60	−	−
35	RIA3_1_31^†^	32	1.04	0.41	0.53, 1.7	0.70
36	RIA5_0_32^†^	34	1.38	0.54	−	−
37	RIA4_1_33^†^	34	0.94	0.37	0.70, 2.1	0.56
38	RIA6_0_34^†^	36	1.24	0.49	0.34, 1.2	0.99
39	RIA5_1_35^†^	36	0.84	0.33	0.92, 2.6	0.46
40	RIA2_1_36^†^	31	1.16	0.45	0.41, 1.4	0.86
41	RIA1_2_37^†^	31	0.79	0.31	1.10, 3.0	0.40
42	RIA1_1_38^†^	29	1.29	0.51	−	−
43	RIA0_2_39^†^	29	0.87	0.34	0.83, 2.4	0.49
44	RIA2_1_40^†^	42	1.16	0.45	0.41, 1.4	0.86
45	RIA1_2_41^†^	42	0.79	0.31	1.10, 3.0	0.40
46	RSA1_0_43^†^	7	1.73	0.68	−	−
47	RSA0_1_44^†^	7	1.17	0.46	−	−
48	RSA2_0_45^†^	46	1.55	0.61	−	−
49	RSA1_1_46^†^	46	1.05	0.41	0.51, 1.6	0.88
50	RSA3_0_47^†^	48	1.40	0.55	−	−
51	RSA2_1_48^†^	48	0.95	0.37	0.68, 2.0	0.71
52	RSA4_0_49^†^	50	1.26	0.49	−	−
53	RSA3_1_50^†^	50	0.85	0.33	0.89, 2.5	0.58
54	RSA5_0_51^†^	52	1.13	0.44	0.43, 1.4	1.01
55	RSA4_1_52^†^	52	0.77	0.30	1.17, 3.1	0.47
56	RSA1_1_53^†^	47	1.05	0.41	0.51, 1.6	0.88
57	RSA0_2_54^†^	47	0.72	0.28	1.40, 3.6	0.41

*The lengths of the first 7 arteries are based on magnetic resonance angiography on a healthy female (25 years old, 172 cm, 71 kg). The reference pressure is assumed to be 10 mmHg. The position of the centerline **G**(s) along each of these 7 arteries is also available as Supplementary Material. Other data are from Mynard and Smolich (2015). **l**: length of the artery; 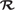: constant radius of the lumen along the artery; R_0_, **R_1_**: resistances in the arteriolar compartments following terminal arteries (see Equation 19); **C_1_**: peripheral compliance associated to the arterioles.*

*^†^Artery considered in the 1D model, but not segmented and thus not included in the BCG computation.*

#### Junctions Matching Conditions

The equations established so far are used to describe the blood flow in each individual artery considered in the model. They need to be completed by interfacing equations at the nodes between these different arteries. In our model, these junctions between vessels are treated as discontinuities, where mass and total pressure are conserved (Formaggia et al., 2006; Alastruey et al., 2012). In the case of splitting flow, using the subscript *m* for the distal end of the mother vessel, *d1* and *d2* for the proximal end of the first and the second daughter vessel, respectively, we can write:


$$\{\begin{array}{cc} Q_{m}(t)=Q_{d1}(t)+Q_{d2}(t) & \left(17\right)\\ P_{m}(t)+\frac{1}{2}\text{ρ}U_{m}{\left(t\right)}^{2}=P_{d1}(t)+\frac{1}{2}\text{ρ}U_{d1}{\left(t\right)}^{2} & \\ =P_{d2}(t)+\frac{1}{2}\text{ρ}U_{d2}{\left(t\right)}^{2} & \left(18\right) \end{array}$$

#### Peripheral Circulation and Venous Return

**FIGURE 3:**
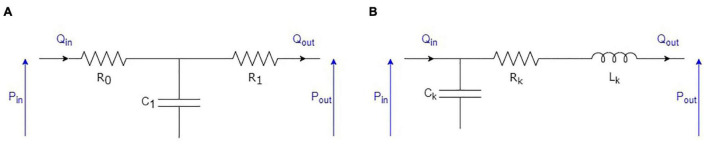
Electrical equivalent to the 0D representation of the elements following the arterial circulation: **(A)** RCR model used for arterioles; **(B)** RLC model used for capillaries, venules, and veins.

The 1D model of the flow in blood vessels described so far is based on assumptions, such as blood being a Newtonian fluid, that are only valid when the diameter of blood vessels is sufficiently large, compared to the size of red blood cells. Thus, as mentioned previously, we limit this 1D model to only 55 arteries in the systemic circulation and 57 arteries in the pulmonary circulation (see Figure 1). Once the most peripheral arteries (so called “terminal branches,” visible in Figure 1, as well as in Tables 2, 3) are reached, a 0D strategy, described in Figure 3 and detailed below, is adopted to model the blood circulation back to the heart. All the parameters defining the 0D compartments are assumed to be time-independent, implying that mechanisms of flow control at the microcirculation and venous levels are not modeled, as it is usually the case in the literature.

##### Arterioles

The junction between the terminal arteries of the 1D models and the following arterioles is modeled using the common RCR approach (see Figure 3A). To each terminal artery, we associate an arteriolar compartment. We note *R*_0_ the resistance used to minimize high frequency reflections at the interface between the 1D and the 0D domains (van de Vosse and Stergiopulos, 2011), *R*_1_ the arteriolar resistance, and *C*_1_ the arteriolar compliance. Consequently, in the arteriolar compartment at the end of each terminal artery, we have the following relationship between input (*Q*_*in*_, *P*_*in*_) and output (*P*_*out*_) variables (Formaggia et al., 2006; Alastruey et al., 2012):


(19) $$\begin{array}{l} Q_{in}(t)(1+\frac{R_{0}}{R_{1}})+C_{1}R_{1}\frac{dQ_{in}(t)}{dt}\\ \;=\frac{P_{in}(t)-P_{out}(t)}{R_{1}}+C_{1}\frac{dP_{in}(t)}{dt} \end{array}$$

For each terminal artery, the numerical values of *R*_0_, *R*_1_, and *C*_1_ are adapted from the literature (Liang et al., 2009a; Mynard and Smolich, 2015) (see Table 2 for the systemic arterioles and Table 3 for the pulmonary arterioles). In the case of the pulmonary arteries, *R*_0_ is evaluated as $R_{0}=\frac{\rho \,c_{0}}{A_{0}}$ at the distal end of the terminal arteries, with *c*_0_ the pulse wave velocity at the reference pressure (Alastruey et al., 2008) (see Section 3 of the Supplementary Material).

##### Capillaries, Venules, and Veins

The rest of the systemic circulation is modeled using RLC compartments (see Figure 3B). In the systemic circulation (see Figure 1), arterioles above and below the heart converge to two different capillary compartments. We thus assume that the output pressures in all the upper (respectively, lower) arteriolar segments and the input pressure of the upper (respectively, lower) capillary compartment are equal. In terms of flow, it also means that the sum of the output flows of all the upper (respectively, lower) arteriolar segments are equal to the input flow of the upper (respectively, lower) capillary compartment. Then, each of these two capillary compartments is followed successively by one venule compartment, one vein compartment, and one vena cava compartment, as in Liang et al. (2009a). In the pulmonary circulation, all the arterioles simply converge to one venous compartment.

In each of these different compartments, we can write two equations to relate the input (*Q*_*in*_, *P*_*in*_) and output (*Q*_*out*_, *P*_*out*_) variables:


$$\{\begin{array}{cc} C_{k}\frac{dP_{in}(t)}{dt}=Q_{in}(t)-Q_{out}(t) & \left(20\right)\\ L_{k}\frac{dQ_{out}(t)}{dt}=P_{in}(t)-R_{k}Q_{out}(t)-P_{out}(t) & \left(21\right) \end{array}$$

where the numerical values of *C_k_*, *L_k_*, and *R_k_* are given in Table 4 for each compartment of the systemic and pulmonary distal circulations.

**TABLE 4:** Values of the parameters used in the compartments of the 0D systemic and pulmonary peripheral circulations.

		Upper body	Lower body	Pulmonary circulation
	*R*_2_ (mmHg.s.ml^–1^)	0.97	0.29	−
Capillaries	*L*_2_ (mmHg.s^2^.ml^–1^)	0.003	0.003	−
	*C*_2_ (ml.mmHg^–1^)	0.03	0.1	−
	*R*_3_ (mmHg.s.ml^–1^)	0.14	0.04	−
Venules	*L*_3_ (mmHg.s^2^.ml^–1^)	0.001	0.001	−
	*C*_3_ (ml.mmHg^–1^)	0.5	1.5	−
	*R*_4_ (mmHg.s.ml^–1^)	0.03	0.009	0.005
Veins	*L*_4_ (mmHg.s^2^.ml^–1^)	0.0005	0.0005	0.0005
	*C*_4_ (ml.mmHg^–1^)	15.0	75.0	17.23
	*R*_5_ (mmHg.s.ml^–1^)	0.0005	0.0005	−
Vena cava	*L*_5_ (mmHg.s^2^.ml^–1^)	0.0005	0.0005	−
	*C*_5_ (ml.mmHg^–1^)	5.0	15.0	−

***R_k_**: equivalent viscous resistance coefficient (mmHg.s.ml**^–^**^1^); **L_k_**: equivalent fluid inertia coefficient (mmHg.s^2^.ml^–1^); **C_k_**: equivalent elastic wall compliance coefficient (ml.mmHg^–1^). Data for the systemic circulation are from Liang et al. (2009a), while data for the pulmonary circulation are adapted from Sun et al. (1997).*

In addition, we add one venous valve in both the upper and lower systemic circulation, between the venous and venae cavae compartments (see Figure 1). The pressure drops through these two valves are modeled as those of the cardiac valves in Section “Cardiac Valves,” with: *A*_eff,max_ =  6.0cm^2^, *l*_eff_ = 1.0cm, *K*_*vo*_ = 40.0mmHg^−1^.s^−1^, *K*_*vc*_ = 40.0mmHg^−1^.s^−1^, and △*P*_close_ = 3mmHg (Mynard et al., 2012).

### Simulated Ballistocardiography Signal

Our nomenclature and the choice of positive directions for the multidimensional BCG are in agreement with the recommendations of the Committee on Ballistocardiographic Nomenclature and Conventions (Scarborough et al., 1956), that is: *x* is the lateral (side to side) component, oriented from left to right; *y* is the longitudinal (caudo-cranial) component, oriented from the feet to the head; and *z* is the anteroposterior (ventro-dorsal) component, oriented from the stomach to the back (see Figure 1).

In our model, it is assumed that the BCG signal is caused solely by movements of blood in the circulatory system. Other effects such as muscle contractions, including contraction of the myocardium, and movements of the body, including respiratory movements, are neglected.

Based on this assumption, it is possible to apply different methods to compute the movements of the body center of mass induced by the cardiovascular activity, i.e., the BCG signal. Some approaches rely on the conservation of momentum (Auslander et al., 1972), others are based on instantaneous distribution of blood mass in the circulatory system (Starr and Noordergraaf, 1967; Guidoboni et al., 2017), while some other simplified models rely only on the blood pressure gradient in the ascending and descending aorta (Yousefian et al., 2019). So far, almost all these methods have focused only on the simulation of the BCG signal on the longitudinal axis.

Here, the simulated BCG signal is computed on the three cardinal axes, based on the knowledge of blood flow between a list of compartments and the tridimensional positions of these compartments in the reference frame of the body (see Section “Anatomical and Physiological References”). In our case, the list of compartments includes the cardiac chambers as well a collection of 0.5-cm elements resulting from the discretization of the arteries, whose positions are obtained from MR angiography. We number these compartments from *1* to *N*. Under the common approximation that only first-order terms may be conserved (Noordergraaf, 1967), the conservation of momentum gives the following relationship:


(22) $$W_{b}\,{\text{BCG}}_{\mathrm{vel}}\,\left(t\right)=W_{b}\,\left(\begin{array}{c} {\dot{x}}_{C\,o\,M}\,\left(t\right)\\ {\dot{y}}_{C\,o\,M}\,\left(t\right)\\ {\dot{z}}_{C\,o\,M}\,\left(t\right) \end{array}\right)=-\rho \,\sum_{j=1}^{N}Q_{j}\,\left(t\right)\,\left({\text{G}}_{j}-{\text{G}}_{\text{u}\,\text{p}\,\left(j\right)}\right)$$

with *W_b_* the body mass of the subject, **BCG_vel_** the velocity BCG signal (*x*_*CoM*_,*y*_*CoM*_,*z*_*CoM*_) the position vector of the center of mass of the body, up(*j*) the index of the compartment immediately upstream of the j-th compartment when blood is flowing in the physiological direction, *Q*_*j*_(*t*) the blood flow rate at the input of the j-th compartment, and **G**_*j*_ the position vector of the center of mass (approximated as the geometrical center) of the j-th compartment.

Then, the acceleration BCG (***BCG*_**acc**_**) and the position BCG (***BCG*_**pos**_**) signals can be obtained by derivation and integration of ***BCG*_**vel**_**, respectively. As recently suggested, the BCG phenomena can also be expressed in terms of kinetic energy (Hossein et al., 2019):


(23) $$B\,C\,G_{\text{kin}}\,\left(t\right)=\frac{1}{2}\,W_{b}\,{||{\text{BCG}}_{\mathrm{vel}}\,\left(t\right)||}^{2}$$

Previous studies have shown that the frequency content of the experimental acceleration BCG signal is in the range 0–40 Hz (Strong, 1970), even though they were focused only on the longitudinal axis. Others have estimated that a low-pass filter at 25 Hz does not significantly affect time-interval measurements (Gómez-Clapers et al., 2013). In practice, low-pass filters at 25 or 35 Hz are commonly used (Inan et al., 2009b; Hossein et al., 2019) and result in a trade-off between the desire to keep as much of the information contained in the signal as possible and the one to remove noise. It is important to highlight the fact that the BCG signal certainly experiences a natural dampening during transmission from the different sources toward the sensors. Depending on the types of body tissues that the vibratory signals go through, the human body is supposed to act as a low-pass filter, whose characteristics are difficult to evaluate. In this study, the theoretical acceleration BCG signal is transformed using low-pass filters and the impact of the cutoff frequency is evaluated at 25 and 40 Hz. In both cases, all the related signals and metrics are computed based on these new filtered signals, in order to compare them with the unfiltered theoretical results.

### Anatomical and Physiological References

This section describes the way numerical parameters are evaluated for the model described up to this point. This computational model is based on the reference case of a healthy young subject, described in Section “Healthy Young Case,” from which modifications are introduced to evaluate the impact of some specific parameters on the BCG signal. In particular, we are interested in cardiovascular modifications occurring during healthy aging, as described in Section “Model of Healthy Aging.” Unless otherwise stated, a heart rate of 70 bpm is assumed, and the results of the computations are meant to represent the cardiovascular status of an adult without concomitant cardiac or arterial diseases. Since significant differences in aortic flow and pressure waves are known to occur even among healthy individuals (Murgo et al., 1980), the main interest of the overall model is to study a typical example and how some output metrics evolve as a function of input parameters, rather than fitting exactly the different curves to experimental data.

#### Healthy Young Case

##### Systemic Arteries and Arterioles

The choice of the 55 arteries used in the 1D model of the systemic circulation originates from the simplified arterial network proposed by Westerhof et al. (1969). They initially gave characteristic parameters corresponding to a healthy subject with a height of 175 cm and a body mass of 75 kg, assuming elastic tapering.

In our reference case of a healthy young adult, the lengths *l*, radii *R*_0_(*s*) (and thus, cross-sectional areas *A*_0_(*s*)), and positions **G**(*s*) of these 55 systemic arteries in the reference frame of the body are obtained from whole-body MR angiography data on a healthy young subject (25-year-old female, height: 172 cm, weight: 71 kg). Segmentation is performed using the open-source software 3DSlicer (The Slicer Community, version 4.11.20200930) and the complete supporting dataset is available as Supplementary Material (see Section 1 of the Supplementary Material). Table 2 presents simplified anatomical data extracted from this segmentation, including the lengths, proximal radii, and distal radii of all these arteries at the reference pressure *P*_*ref*_, assumed to be 70 mmHg. We observe a good agreement between the geometry of the segmented vessels and data from the literature (Liang et al., 2009a).

Among the 55 arteries listed for the systemic circulation in Table 2, a total of 42 arteries are clearly visible on the MR angiography pictures and were thus segmented. Eight arteries of the forearm are not visible on the imaged volume, while the intercostal arteries and 4 additional arteries of the abdomen are poorly visible. For these arteries, segmentation is not possible, so the dataset provided by Liang and colleagues is used instead to express their lengths and radii (Liang et al., 2009a). For these 13 unsegmented small arteries (clearly mentioned in Table 2), we can thus only compute the blood pressure and the flow rate, but not the contribution to the BCG signal. However, we expect this contribution to be negligible due to the small amplitude of blood flow rate variation in these arteries (radius lower than 3 mm). The same dataset from Liang and colleagues also serves as a reference to extrapolate the geometry of two arteries (left and right subclavian II) that are only partially visible on the MR angiography pictures.

For each artery, empirical laws (see Section 3 of the Supplementary Material) are used to compute the pulse wave velocity *c*_0_(*s*), the coefficient β(*s*), and the viscous parameter Γ(*s*), based on the radius ℛ(*s*) extracted from the segmentation of the systemic arterial tree.

##### Pulmonary Arteries and Arterioles

The 57 arteries used in the 1D model of the pulmonary circulation are the ones suggested by Mynard and Smolich (2015), based on a fractal representation of the pulmonary arterial tree (Qureshi et al., 2014) with a length/radius ratio of 2.55 and the following constitutive relationships:


(24) $${ℛ}_{m}^{\xi }={ℛ}_{d\,1}^{\xi }+{ℛ}_{d\,2}^{\xi }$$

(25) $$\gamma =\frac{{ℛ}_{d\,2}^{2}}{{ℛ}_{d\,1}^{2}}$$

where the fractal exponent ξ = 2.76 and the asymmetry ratio γ = 0.43. The subscripts are the same as those introduced earlier in the case of splitting branches: *m*, for the mother vessel; *d*_1_, for the first daughter vessel; and *d*_2_, for the second daughter vessel. Moreover, unlike the case of the systemic arteries, we assume the radii of the pulmonary arteries to be constant along their length.

In the pulmonary circulation, only the 7 first arterial branches were segmented from the whole-body MR angiography data on the healthy young subject, corresponding thus to the first three generations of the fractal tree. The lengths of these 7 arteries were updated accordingly (i.e., replaced by the values from MR angiography), while their radii were kept at the values suggested by Mynard and Smolich (2015), based on Equations 24, 25. This also means that only these 7 pulmonary arteries were included in the computation of the BCG signal. The impact of these assumptions is expected to be very small, since previous studies have shown that the contribution of the pulmonary arteries to the BCG signal is minor (Noordergraaf et al., 1959). This is also confirmed here in the results presented in Section “General Features of the Simulated Ballistocardiography Signals.”

Table 3 summarizes the data used in the pulmonary arterial circulation at the reference pressure *P*_*ref*_, assumed to be 10 mmHg. The position of the 7 first pulmonary arteries is also available as Supplementary Material.

For each artery, empirical laws are used to compute the pulse wave velocity *c*_0_(*s*) and the coefficient β(*s*) (see Section 3 of the Supplementary Material). The viscous parameter Γ(*s*) is supposed to be constant: Γ(s) = 1.5× 10^−3^ m.s.*mmHg* (Mynard and Smolich, 2015).

##### Heart

Table 1 presents the numerical values chosen for parameters related to the cardiac chambers, based on data published by Liang et al. (2009a) and adapted for a heart rate of 70 bpm. It also includes the numerical values chosen for the cardiac valves, based on data published by Mynard and Smolich (2015). In the case of the pulmonary and aortic valves, we have adapted the value of *A*_*eff,max, CV*_ to match the cross-sectional area of the main pulmonary artery and of the proximal ascending aorta, respectively. Since we assume no cardiac or valve pathologies in the reference healthy case, all the cardiac valves can open and close entirely.

##### Other 0D Compartments

The numerical values of the equivalent viscous resistance, fluid inertia, and elastic wall compliance parameters used in the compartments of the 0D peripheral circulation are presented in Table 4. They are taken from Liang et al. (2009a) for the systemic (lower and upper body) circulation and adapted from Sun et al. (1997) and Mynard and Smolich (2015) for the pulmonary circulation.

Because of the closed-loop nature of this model, the total amount of blood does not change during the simulation, which makes it even more important to use suitable initial conditions in terms of pressure in the different compartments. In this case, the necessary adaptations in terms of blood volume are achieved by changing the initial pressure in the venous compartments, because of their high compliance. The total blood volume (*TBV*, in liters) is assumed to be given by Nadler’s equation (Nadler et al., 1962), here in the case of female subjects:


(26) $$T\,B\,V=0.3561\,H_{b}^{3}+0.03308\,W_{b}+0.1833$$

with *H_b_*, the body height (in meters) and *W_b_*, the body mass (in kilograms) of the subject. For the particular subject considered in the healthy young case, Equation 26 gives *TBV* = 4440 ml. We also assume that 27.5% of *TBV* corresponds to the stressed volume causing positive pressure in the different compartments (Magder, 2016).

#### Model of Healthy Aging

Healthy aging is simulated based on the healthy young case described previously, at a constant heart rate of 70 bpm, with some age-induced modifications summarized in Table 5 and in the absence of any concurrent pathology. In particular, the impact of age on the BCG signal is evaluated by comparing typical conditions decade per decade for subjects between 20 and 80 years old.

**TABLE 5:** Values used in the model of healthy aging.

Age (years)	20	30	40	50	60	70	80
*c*_0_ (m.s^–1^)	4.91	5.33	5.91	6.66	7.57	8.65	9.89
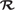 _0_ (cm)	1.18	1.23	1.27	1.31	1.36	1.40	1.45
*C*_*per*_ (ml.mmHg^–1^)	3.08	2.75	2.42	2.09	1.76	1.43	1.10
*E*_*A*,*LV*_ (mmHg.ml^–1^)	2.71	2.79	2.86	2.93	3.00	3.07	3.15
*E*_*B*,*LV*_ (mmHg.ml^–1^)	0.096	0.104	0.113	0.121	0.129	0.138	0.146
*T*_*vrp*_ (s)	0.132	0.146	0.160	0.174	0.188	0.202	0.216

*c_0_ and 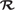
_0_: pulse wave velocity and radius, respectively, evaluated at the middle of the ascending aorta; C_per_: total systemic peripheral compliance (sum of all C_1_ in Table 2, and C_2–3_ in Table 4); E_A, LV_ and E_B, LV_: amplitude and baseline elastance of the left ventricle, respectively; T_vrp_: duration of the ventricular relaxation.*

Since changes in distensibility of muscular arteries are relatively small (Nichols et al., 2011), arterial stiffening with age is simulated only in the different segments of the aorta. In each of these arterial segments, the pulse wave velocity (which, as mentioned in Section 3 of the Supplementary Material is used to calculate the coefficient β(*s*) of the segment) is modified from its baseline value $c_{0}^{25\,y}$, for a subject aged 25. To do so, we adapt the regression equation given in The Reference Values for Arterial Stiffness’ Collaboration (2010) so that it is centered around the age of the reference healthy young subject:


(27) $$c_{0}^{\text{age}}=c_{0}^{25\,y}+4.15\times 10^{-2}\,\left(\text{age}-25\right)+8.30\times 10^{-4}\,{\left(\text{age}-25\right)}^{2}$$

with age, the simulated age in years.

To remain coherent with the coordinates of the arterial segments measured by MR angiography, progressive lengthening of the aorta with age is not simulated. This approximation should not have a large impact on the results, since this age-related elongation is relatively small compared to the increase in pulse wave velocity (Hickson et al., 2010). However, for each of the aortic segments, we simulate a progressive widening using the formula suggested by Charlton et al. (2019):


(28) $${ℛ}_{0}^{\text{age}}={ℛ}_{0}^{25\,y}\,\left(9.09\times 10^{-1}+3.65\times 10^{-3}\times \text{age}\right)$$

with ${ℛ}_{0}^{25y}$, the baseline radius value for the reference subject, aged 25, and ${ℛ}_{0}^{\mathrm{age}}$, its value at the simulated age.

The resulting values of radii in all the aortic segments agree with observations in healthy subjects of various ages (Hickson et al., 2010; Dietenbeck et al., 2021).

Systemic vascular resistance remains relatively unchanged with age in healthy subjects (Redfield et al., 2005; Wooten et al., 2021). Thus, we keep constant the resistances in all the 0D elements of the systemic circulation. The same is done for the pulmonary circulation.

Peripheral (arterioles, capillaries, and venules) systemic compliances are reduced with age as in Charlton et al. (2019):


(29) $$C_{k}^{\text{age}}=C_{k}^{25\,y}\,\left(12.84\times 10^{-1}-11.36\times 10^{-3}\times \text{age}\right)$$

with *k* ∈ {1,2,3} (see Tables 2–4).

Age-related ventricular and arterial stiffening occur simultaneously so that ventricular-vascular coupling is relatively unchanged (Chen et al., 1998). Since the general expressions of the normalized varying elastance curves *e*_*CC*_(*t*) are relatively unaffected by age (Senzaki et al., 1996), we model this ventricular stiffening by an increase in $E_{A,L\,V}^{\text{age}}$ and $E_{B,L\,V}^{\text{age}}$ (amplitude and baseline elastance of the left ventricle at a given age, respectively), based on their values at 25 years old in the healthy young case (Redfield et al., 2005):


(30) $$E_{A,L\,V}^{\text{age}}=E_{A,L\,V}^{25\,y}+7.20\times 10^{-3}\times \left(\text{age}-25\right)$$

(31) $$E_{B,L\,V}^{\text{age}}=E_{B,L\,V}^{25\,y}+8.41\times 10^{-4}\times \left(\text{age}-25\right)$$

The ability of myocardial cells to quickly contract and relax is affected by age (Lakatta et al., 1975; Nakou et al., 2016), leading in particular to a marked increase in the relaxation duration (Nichols et al., 2011). Here, we model this phenomenon similarly in the left and right ventricles, assuming that the duration of the ventricular relaxation increases with age in a similar way as the left ventricular isovolumic relaxation time (Villari et al., 1997). While Nikitin et al. (2005) found a stabilization of the isovolumic relaxation time after 60 years old, this was not observed in all studies (Peverill, 2019). To simplify the relationship of relaxation time with age and avoid survival bias, we suggest a linear relationship:


(32) $$T_{v\,r\,p}^{\text{age}}=T_{v\,r\,p}^{25\,y}\,\left(7.50\times 10^{-1}+1.00\times 10^{-2}\times \text{age}\right)$$

with $T_{v\,r\,p}^{25\,y}$, the duration of the ventricular relaxation for the reference healthy subject, aged 25, and $T_{v\,r\,p}^{\text{age}}$, its value at the simulated age.

### Summary

The input parameters of this computational model include the geometry, the position, and the elastic and viscous coefficients (β and Γ, respectively) of the different arteries, the parameters defining the fractal structure of the pulmonary arterial tree (length/radius ratio, ξ, γ), the characteristic parameters of the peripheral circulations compartments (*R_k_*, *L_k_*, *C_k_*), the characteristic parameters of the cardiac and venous valves (*A*_*eff,max*_, *l*_*eff*_, *K*_*vo*_, *K*_*vc*_, △*P*_close_, △*P*_open_), as well as the parameters defining elasticity in the cardiac chambers (minimum elastance, amplitude of elastance, duration of contraction and relaxation).

Geometrical information from 55 systemic (see Table 2) and 57 pulmonary (see Table 3) arteries is used to compute all the parameters necessary to solve, for each artery, Equations 15, 16 based on a 1D representation of each of these two arterial trees and junction matching conditions described by Equations 17, 18. The 1D representations of both arterial trees are coupled using 0D elements as described in Figure 1. These 0D elements include a model of the peripheral circulation (see Table 4) described by Equations 19–21 and a time-varying elastance model of the heart (see Table 1) based on Equation 3.

This system of equations is numerically solved, using a numerical procedure written in Wolfram Mathematica 11.2, until the signals generated by two consecutive heartbeats overlap. Independence of the results toward discretization size has been checked for all the cases presented in this paper.

After this resolution, blood flow rates and pressures are available in all the compartments previously described (each of the 0D elements and each discretized element of the considered arteries).

Equation 14 is then used to compute the corresponding blood volume in each discretized element of the considered arteries, while Equation 1 is used for the cardiac chambers. Finally, the velocity BCG signal is computed using Equation 22, based on the conservation of momentum between the compartments whose coordinates have been measured by whole-body MR angiography, i.e., the cardiac chambers, each discretization element of the first 7 arteries in the pulmonary circulation, and each discretization element of the 42 arteries that have been segmented in the systemic circulation.

## Results and Discussion

### Hemodynamics in the Healthy Case

**FIGURE 4:**
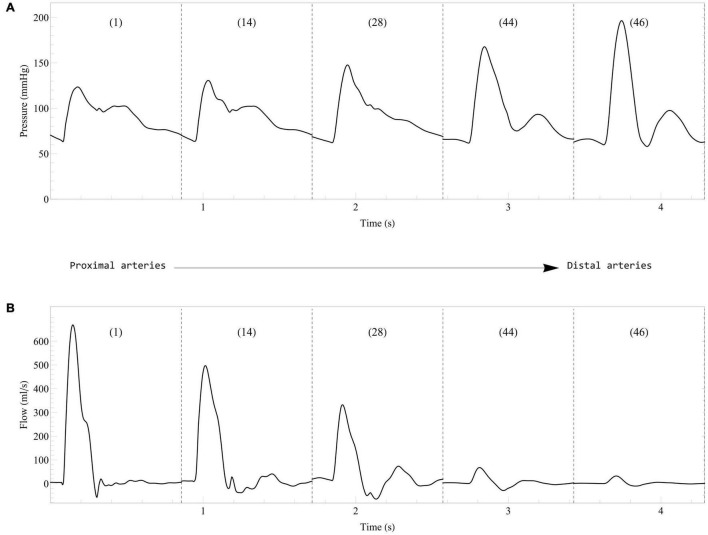
Evolution of blood pressure **(A)** and flow rate **(B)** in some selected systemic arteries. Results from the computational model in the healthy case are displayed from the most proximal to the most distal arteries. The arteries are numbered according to the list in Table 2: 1, Ascending aorta; 14, Aortic arch II; 28, Abdominal aorta I; 44, L. external iliac; 46, L. femoral.

In the healthy young case described in Section “Healthy Young Case,” the computational model displays results in agreement with normal physiology (Klabunde, 2011), with a higher end-diastolic volume in the right ventricle (135 ml) than in the left ventricle (119 ml) (Hudsmith et al., 2005). The left ventricle stroke volume and ejection fraction are 76 ml and 63%, respectively. The left ventricle diastolic function is also normal with a peak early filling rate of 415 ml/s and a E/A ratio of 1.65. Figure 4 shows the evolution of blood pressure and flow rate in some selected arteries. As expected, moving away from the heart, we observe a delay in pressure signals, an increase in systolic pressures, a decrease in diastolic pressures, and a decrease in the amplitude of blood flow rates (van de Vosse and Stergiopulos, 2011). In addition, systolic/diastolic pressures in both arterial trees are also within the normal range of physiology with 123/63 mmHg in the ascending aorta (see Figure 4A) and 27/14 mmHg in the main pulmonary artery.

### General Features of the Simulated Ballistocardiography Signals

**FIGURE 5:**
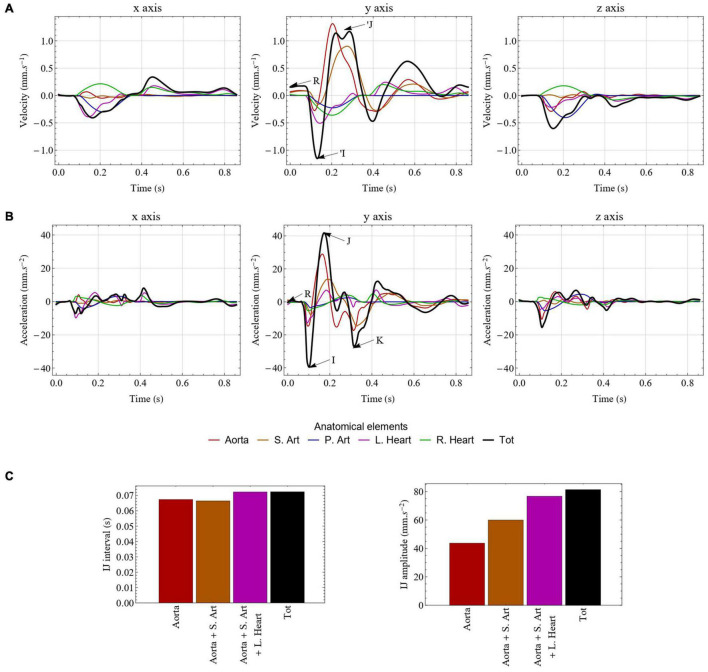
Unfiltered velocity **(A)** and acceleration **(B)** BCG signals generated by the computational model in the healthy case. The contribution of each type of elements is indicated by different colors. The traditional I, J, and K waves are indicated on the *y* axis of the acceleration BCG signal, while R corresponds to the R peak of the ECG and indicates the beginning of ventricular contraction. The ‘I and ’J waves are also indicated on the *y* axis of the velocity BCG signal. **(C)** Impact of taking into account various compartments of the circulatory system on the evaluation of the IJ interval (left) and the IJ amplitude (right). S. Art: systemic arteries (excluding aorta); P. Art: pulmonary arteries; L. Heart: left side of the heart; R. Heart: right side of the heart; Tot: total.

The unfiltered velocity and acceleration BCG signals generated by the computational model in the healthy reference case are displayed in Figures 5A,B, respectively, on the three cardinal axes. In addition, Figure 5 also represents the contribution of the different elements to the BCG signal: the aorta, the other systemic arteries, the pulmonary arteries, the left heart, and the right heart.

As in previous computational models of BCG (Noordergraaf et al., 1959; Kim et al., 2016; Guidoboni et al., 2017; Yousefian et al., 2019), the classical shape and amplitude of ***BCG*_**acc**_** on the longitudinal (*y*) axis is well reproduced, with a very clear IJK complex following systole, while the pre-ejection upward H wave is not visible. More details regarding the comparison with experimental signals are given in Section 5 of the Supplementary Material. We observe that the downward I wave is mainly generated by the acceleration of blood from the left ventricle to the ascending aorta, while the upward J wave is associated with the changing direction of the pulse wave as it goes from the ascending aorta to the descending aorta. Finally, the downward K wave mostly corresponds to the decrease of blood flow rate and the reflection of the pulse wave at the periphery of the systemic arterial tree.

The absence of the H wave on the simulated BCG signals indicates that the 1D elements considered are not the one causing this particular wave. Based on signals recorded during complete heart block, several authors have already been able to associate this wave to the atrial systole (Nickerson, 1949; De Lalla et al., 1950), and especially the right atrial systole (Starr and Noordergraaf, 1967). This means that the H wave may be caused by the pressure wave generated by the right atrium and traveling through the vena cavae. Such a hypothesis is supported by the fact that Auslander and colleagues managed to reproduce an H wave with an early model including the large systemic veins (Auslander et al., 1972). Since the inferior vena cava carries more blood than the superior one, and since arches and bifurcations in the superior vena cava cause the upward blood flow to quickly decelerate, it could explain the fact that the experimentally observed H wave is in the upward direction.

The results displayed in Figure 5B show that the waves occurring after the K wave on the longitudinal axis of ***BCG*_**acc**_**, sometimes called diastolic waves, are not only caused by ventricular filling, but are also strongly influenced by blood flow in the systemic arteries, including the aorta. This is in agreement with previous observations (Noordergraaf et al., 1959). However, in the posteroanterior (*z*) and lateral (*x*) components of ***BCG*_**vel**_**, the diastole is much less affected by the systemic circulation and better reflects the two phases of ventricular filling, as depicted in Figure 5A.

Figure 5B shows that the aorta is the largest contributor to the longitudinal ***BCG*_**acc**_** signal, but that the other systemic arteries have a contribution that is far from negligible. The same holds true for the cardiac chambers of the heart, and especially the left heart, even though their impact is much smaller. This highlights that the results provided by computational models of longitudinal ***BCG*_**acc**_** based on the sole evaluation of blood flow in the aorta (Kim et al., 2016) are missing a non-negligible part of the actual movements of the body induced by cardiovascular activity. Even if such aorta-based models may provide relatively good estimates of time intervals between the different waves, they cannot accurately reflect metrics related to the amplitude of the BCG signal. Besides this, a more complete model, such as the one developed in this work, offers a better understanding of the complex interplays that may occur through the circulatory system. Indeed, even though the direct impact of the pulmonary circulation appears to be relatively small, it may cause significant changes in the systemic circulation and thus indirectly impact the BCG signal.

The influence of the various compartments on the BCG signal is further evaluated in Figure 5C, where the IJ amplitude and the time interval between the I and J peaks are computed based on the inclusion of different compartments in the BCG calculation (without modifying the model of blood circulation). While the sole contribution of the aorta gives a very reliable IJ time interval, it should be noted that about half of the IJ amplitude originates from other compartments than the aorta.

It is also worth mentioning the fact that the blood flow in the aorta, which plays a key role in the generation of the BCG signal, is strongly affected by the update of the ζ_*i*_ parameters. Indeed, the hypothesis that the velocity profile is parabolic in the first iteration is far from being verified in the aorta (and in some arteries downstream). This explains the differences in some BCG metrics between the first and second iteration of our procedure calculate the ζ_*i*_ parameters (result not presented), thus justifying this iterative approach. Tridimensional ***BCG*_**acc**_** signals have not been extensively studied in the past, but the results presented in Figure 5B have a similar shape and amplitude as experimental records secured several decades ago on healthy subjects in supine position, with devices such as suspended beds (Tannenbaum et al., 1954; Soames and Atha, 1982). In particular, we observe that the longitudinal axis (*y*) of ***BCG*_**acc**_** is the one with the largest amplitude, followed by the anteroposterior axis (*z*) and the lateral axis (*x*). However, records performed in sitting position led to different results, with the amplitudes on all axes being relatively similar (Soames and Atha, 1982). Comparable results have been found using wearable sensors in weightlessness (Hixson and Beischer, 1964; Prisk et al., 2001) and dry immersion (Migeotte et al., 2011). This underlines the necessity to correctly restrain the body when measuring BCG signals in other dimensions than the longitudinal one, as described earlier (Starr and Noordergraaf, 1967), since rolling and pitching movements may strongly affect the measurement of linear accelerations. Measurements of angular velocities confirm this hypothesis (Hixson and Beischer, 1964), as metrics based on kinetic energy have shown similar orders of magnitude in both linear and rotational dimensions (Hossein et al., 2021b). Early computational models have also computed the lateral component of the BCG signal (Starr and Noordergraaf, 1967). They found that the effect on this signal of the changing distribution of blood in the pulmonary circulation is small. Figure 5 indeed suggests that the influence of the right heart and the pulmonary arteries on the *x* component of ***BCG*_**acc**_** and ***BCG*_**vel**_** almost compensate, while the impact of systemic arteries is relatively small.

**FIGURE 6:**
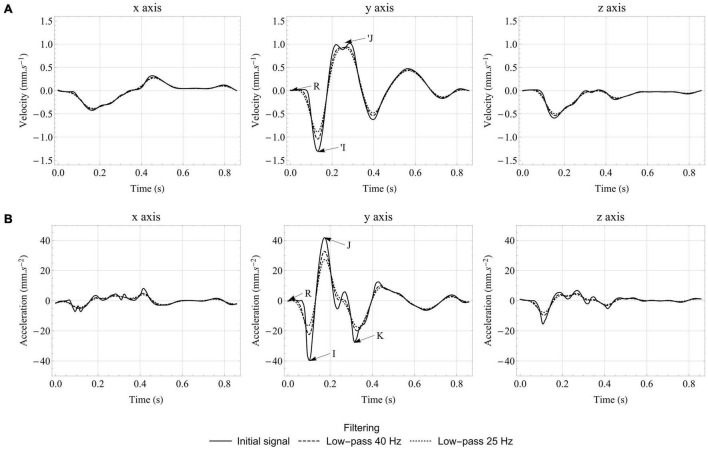
Effect of low-pass filtering on velocity **(A)** and acceleration **(B)** BCG signals generated by the computational model in the healthy case. The traditional I, J, and K waves are indicated on the *y* axis of the acceleration BCG signal, while R corresponds to the R peak of the ECG and indicates the beginning of ventricular contraction. The ‘I and ’J waves are also indicated on the *y* axis of the velocity BCG signal.

The effect of low-pass filtering on the signals generated by the computational model in the healthy case is presented in Figure 6. The amplitude of the different waves is reduced by removing the highest frequencies, but one can still see very clearly the IJK complex on the longitudinal axis of ***BCG*_**acc**_**, even with a low-pass 25 Hz filter. As expected, the acceleration signal is the most impacted by the different levels of filtering, since integration helps preserving the velocity signal. This means that metrics based on ***BCG*_**vel**_** may be less affected by variability in dampening parameters caused by inter-individual differences, especially regarding body composition. However, these metrics may also be less sensitive to changes in the cardiovascular condition.

Interestingly, Figure 6B shows that the relative size of the different peaks is largely affected by filtering. In the unfiltered case, the I wave has a larger amplitude than the K wave. However, the K wave is wider and thus less affected by low-pass filters. With a cutoff frequency at 25 Hz, the amplitude of the I and K waves are approximately the same, which is closer to what is observed experimentally (Scarborough et al., 1953; Starr and Noordergraaf, 1967). Low-pass filtering also reduces the amplitude differences between the three axes of ***BCG*_**acc**_**, making them closer to experimental observations, but not sufficiently to invalidate previous assumptions about the impact of rotations on BCG signals for unrestrained subjects.

### Effect of Healthy Aging

**FIGURE 7:**
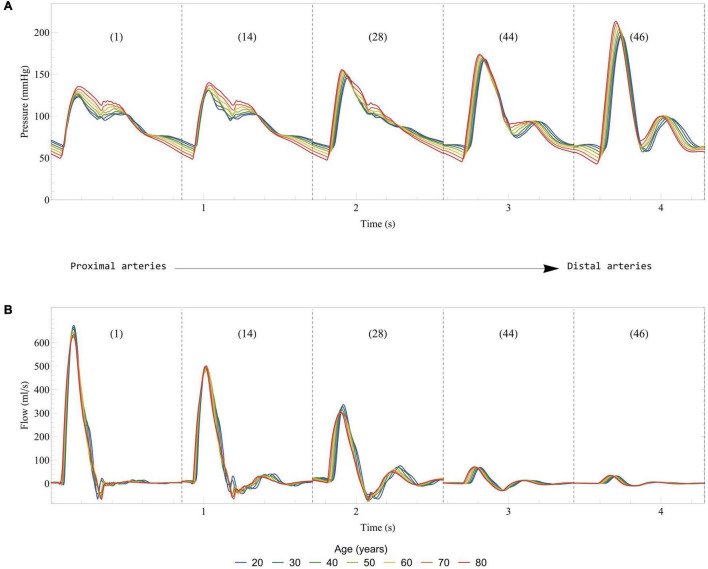
Effect of healthy aging on blood pressure **(A)** and flow rate **(B)** in some selected systemic arteries. Results are displayed from the most proximal to the most distal arteries. The arteries are numbered according to the list in Table 2: 1, Ascending aorta; 14, Aortic arch II; 28, Abdominal aorta I; 44, L. external iliac; 46, L. femoral.

Figure 7 displays some of the hemodynamic results in the systemic arteries in the case of healthy aging. These results are in good agreement with clinical observations (Nichols et al., 2011) and previous numerical models (Liang et al., 2009b; Charlton et al., 2019): a progressive increase of the systolic pressure and slight decrease of the diastolic pressure, a slight decrease in the peak of the aortic flow rate, while the stroke volume (area under the curve of flow rate for the ascending aorta) remains relatively unchanged. We also observe that the diastolic function is altered by aging, as it can be seen by looking at the maximal filling rates of the ventricles during early and late diastole (results not presented), and in particular the decrease of the E/A ratio (maximal filling rate during early diastole versus late diastole).

**FIGURE 8:**
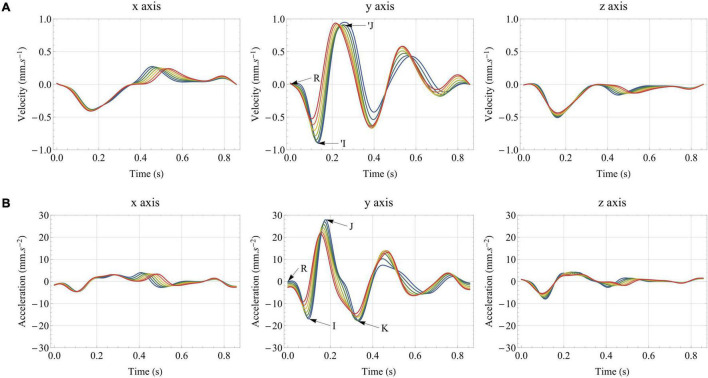
Effect of healthy aging on: **(A)** the tridimensional velocity BCG signal (***BCG*_*vel*_**); **(B)** the tridimensional acceleration BCG signal (***BCG*_*acc*_**). All the results are obtained after low-pass filtering of the acceleration signal at 25 Hz. The traditional I, J, and K waves are indicated on the *y* axis of the acceleration BCG signal, while R corresponds to the R peak of the ECG and indicates the beginning of ventricular contraction. The ‘I and ’J waves are also indicated on the y axis of the velocity BCG signal.

Figure 8 presents the evolution of the tridimensional ***BCG*_**vel**_** and ***BCG*_**acc**_** signals in healthy aging, between 20 and 80 years old. Here, we discuss only the case of low-pass filtering at 25 Hz, but the 40 Hz low-pass and unfiltered results lead to the exact same findings. More generally, all the results presented in the Section “General Features of the Simulated Ballistocardiography Signals,” regarding the relative contribution of the different elements and the effect of filtering, are still valid. On the longitudinal axis of ***BCG*_**acc**_**, we observe a large decrease in the amplitude of the I wave with aging, while the amplitudes of the J and K waves experience a lower decrease, in agreement with previous clinical observations (Scarborough et al., 1953), including longitudinal studies (Starr and Wood, 1961). This result is all the more interesting as an abnormally low amplitude of the IJ complex has been shown to be an indicator of higher chances of developing cardiac diseases (Starr and Wood, 1961). In contrast, the subsequent diastolic waves show an increased amplitude with aging, which agrees with clinical observations made on ***BCG*_**pos**_** with increased pulse wave velocity (Felderhoff and Klensch, 1960). These observations are most probably due to the increased systemic pressure in aging, leading to a slower acceleration and a faster deceleration of blood. Such an interpretation is supported by observations on animal models following occlusion and release of occlusion in the venae cavae (Knoop, 1965), but also by findings following abdominal compression (Scarborough et al., 1953).

The I, J, and, to a lesser degree, the K waves appear earlier in the cardiac cycle for older subjects, in agreement with large scales studies (Scarborough et al., 1953) and recent simplified models looking at the effect of increased arterial stiffness (Yousefian et al., 2019). In contrast, the IJ, IK, and JK time intervals are relatively stable. This slightly differs from the findings of Scarborough and colleagues, who concluded that age could be one factor, among others, for somewhat decreasing values of the IJ time interval (Scarborough et al., 1953). However, it should be noted that we also observe an increase of the left ventricular ejection time (*LVET*) with aging in the simulated cases, which may have an influence on the generation of the different systolic waves, as studied in Section “Pulse Wave Velocity.”

Relatively similar observations can be made on the longitudinal ***BCG*_**vel**_** signal during simulated aging, with a decrease of the amplitude of the first systolic wave (’I), while the amplitudes of the diastolic waves increase. Figure 8 shows that all the waves also occur earlier in the cardiac cycle with aging. The amplitude of the second systolic wave (’J) remains impressively stable and the same holds true for the integral of the ***BCG*_**vel**_** vector over the whole cardiac cycle. To a lesser extent, the integral of *BCG*_kin_ over the whole cardiac cycle, computed based on the ***BCG*_**vel**_** vector and shown to correlate to stroke volume in increased (Hossein et al., 2019) and decreased (Rabineau et al., 2020) contractility settings, is also relatively stable. For more information regarding the relationship between stroke volume and *BCG*_kin_, see also Section “Stroke Volume.”

As shown in Figure 8A, the systolic parts of the *x* and *z* axes of ***BCG*_**vel**_** are left largely unaffected by simulated healthy aging. This is not the case of the diastolic part, which is certainly affected by relatively large changes in left ventricle elasticity and their consequences in terms of ventricular filling.

### Physiological Meaning of the Ballistocardiogram

To better understand the physiology behind the BCG signal, we apply the computational model to a range of input conditions. In addition to the reference case presented in Sections “Healthy Young Case”, we simulate subjects aged from 20 to 80 years old, with a 10-year interval, as well as three values of heart rate (60, 70, and 80 bpm), and three values of stressed volume (corresponding to 25, 27.5, and 30% of the total blood volume computed with Equation 26). Each simulation allows the computation of some physiological parameters (such as the stroke volume, among others) and some BCG parameters. Based on reasonable physiological assumptions, we evaluate the correlation between these BCG-based metrics and these physiological parameters. In particular, the following sections highlight the potential of BCG to monitor indicators of the vascular health (pulse wave velocity) and the systolic function (stroke volume).

#### Pulse Wave Velocity

Based on the physiological origin of the systolic waves on the longitudinal ***BCG*_**acc**_** signal (see Section “General Features of the Simulated Ballistocardiography Signals”), the time intervals between the peaks of these waves can be highly influenced by the pulse transit time along the aorta. This hypothesis is supported by the experimental observation that age is a contributing factor leading to shorter time intervals between the systolic waves (Scarborough et al., 1953), as well as the positive correlation between body size and the durations of these time intervals (Scarborough et al., 1953). Other observations have shown that the amplitude of the K wave was larger in the case of longer descending aortas (Nickerson, 1949). The very high repeatability of the experimental measures of these time intervals, and in particular the one between the peaks of the J and the K waves (Inan et al., 2009b), supports the fact that they are mainly influenced by the characteristics of the aorta (length, cross-sectional area, stiffness). Recent simplified computational models have also provided elements confirming the role of the pulse wave velocity along the aorta in the time interval between these systolic waves (Kim et al., 2016; Yousefian et al., 2019).

Since we conserve the lengths of the different vessels in all the simulations, these parameters can be excluded from the correlation analysis. Instead, we directly analyze the relationship between time intervals measured on the longitudinal ***BCG*_**acc**_** signal and the inverse of the pulse wave velocity. Except for RI and RJ, the raw time intervals correlate relatively poorly with the inverse of the pulse wave velocity (results available in Section 6 of the Supplementary Material). Figure 8 shows indeed that the different peaks of the longitudinal ***BCG*_**acc**_** are not all similarly shifted during aging, and thus increase in pulse wave velocity. In addition, the shift observed in the positions of these peaks is relatively small, while the aortic pulse wave velocity is approximately doubled between 20 and 80 years old. Based on assumptions developed in Section 6 of the Supplementary Material, we suggest the following scaling law to correlate the time interval △*t* with the inverse of the pulse wave velocity:


(33) $$\frac{1}{c_{0,\text{Ao}}}=\kappa_{P\,T\,T}\,\sqrt{\frac{△\,t\,\sqrt{T_{R\,R}}}{A_{0,\text{Ao}}\,L\,V\,E\,T}}=\kappa_{P\,T\,T}\,\tilde{P\,T\,T_{△\,t}}$$

with *c*_*0,Ao*_ the average pulse wave velocity along the aorta, *A*_*0,Ao*_ the average cross-sectional area at pressure *P*_ref_along the aorta, $\tilde{P\,T\,T_{△\,t}}$, measured in s^1/4^.cm^–1^, being equivalent to a pulse transit time after a transformation operated on the △*t* time interval between two events on the BCG signal, and κ_*PTT*_ a proportionality factor measured in s^3/4^.

**FIGURE 9:**
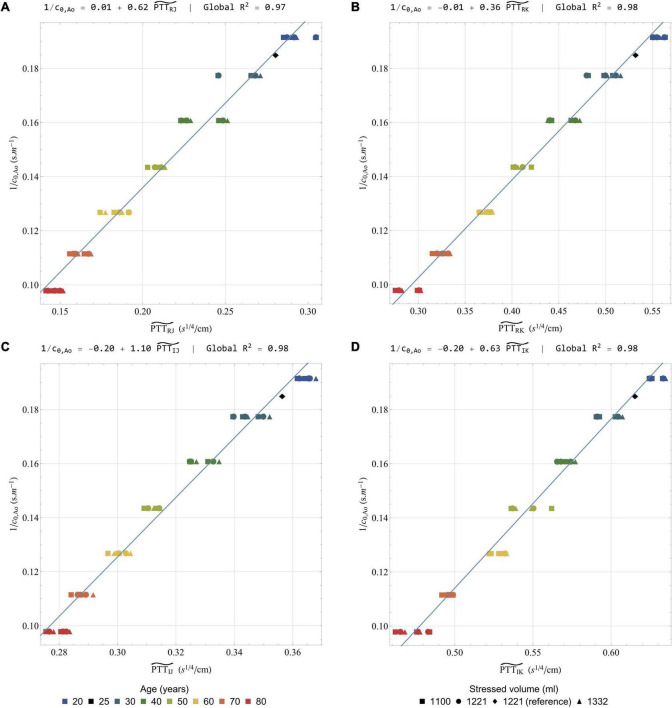
Correlation of the inverse of the pulse wave velocity in the aorta with a selection of rescaled time intervals: **(A)** $\tilde{P\,T\,T_{R\,J}};$ **(B)** $\tilde{P\,T\,T_{R\,K}};$ **(C)** $\tilde{P\,T\,T_{I\,J}};$ and **(D)** $\tilde{P\,T\,T_{I\,K}}$. All the results are obtained after low-pass filtering of the acceleration signal at 25 Hz. In each case, the global linear regression line is indicated together with its equation and the regression coefficient R^2^. Colors indicate different simulated ages, while symbols indicate different stressed volumes. Several points can have the same color and symbol, when they correspond to different heart rates. The healthy young reference case is indicated by a black diamond.

Figure 9 presents the correlation between the inverse of *c*_*0,Ao*_ and a list of corrected time-intervals: $\tilde{P\,T\,T_{R\,J}}$, $\tilde{P\,T\,T_{R\,K}}$, $\tilde{P\,T\,T_{I\,J}}$, and $\tilde{P\,T\,T_{I\,K}}$. Additional correlations are also presented in the Supplementary Material. The linear correlation coefficients are all excellent, with R^2^ systematically over 0.96. Besides this, the equation of the regression line for $\tilde{P\,T\,T_{R\,J}}$ and $\tilde{P\,T\,T_{R\,K}}$ is very close to the form *y* = *Ax* (see Figures 9A,B). This proves the ability of the suggested scaling law to predict changes in pulse transit time, and thus potentially in pulse wave velocity, by taking into account differences of heart rates and mean cross-sections of the aorta.

The use of *A*_*0,Ao*_ and *LVET* in the suggested transformation makes it less straightforward to apply. However, if no MRI or echocardiographic measurement is available, *A*_*0,Ao*_ could be at least estimated, based on parameters such as the age, height, and weight of the subject, among others. With regard to *LVET*, the simultaneous measurement of another cardiac-induced vibration measurement technique may also prove useful, since many studies claim that *LVET* can be measured by seismocardiography (Marcus et al., 2007; Zakeri et al., 2020).

As already described in the literature, low pass filtering within a reasonable range of frequency does not critically affect the position of the different peaks from the longitudinal ***BCG*_**acc**_** signal (Gómez-Clapers et al., 2013). This can be visually checked on the different filtered and unfiltered signals from the healthy young reference case (see Figure 6) and has been verified for all the different simulated cases (supporting results available in Section 6 of the Supplementary Material). The time intervals between these peaks appear then as a reliable metrics that should not be affected by the natural dampening of the BCG signal, and the inter-subject differences in body types.

#### Stroke Volume

**FIGURE 10:**
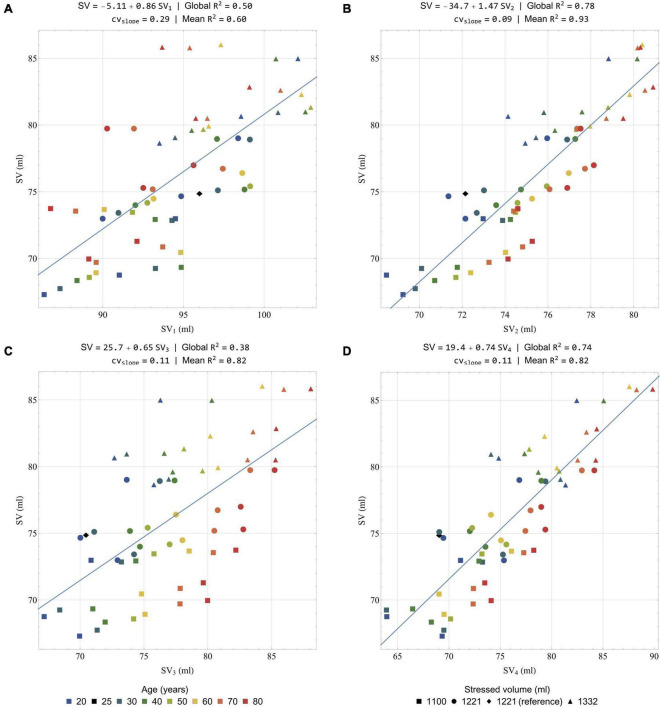
Correlation of actual (i.e., calculated from the solution of the model equations) stroke volume (ordinate) with a selection of estimated stroke volumes (abscissa): **(A)** *SV*_1_ (Equation 34); **(B)** *SV*_2_ (Equation 35); **(C)** *SV*_3_ (Equation 36); and **(D)** *SV*_4_ (Equation 37). All the results are obtained after low-pass filtering of the acceleration signal at 25 Hz. Global R^2^: correlation coefficient computed on the whole dataset; Mean R^2^: mean correlation coefficient computed on the 7 regressions for the 7 simulated ages; *cv*_slope_: coefficient of variability of the slope among the 7 regressions for the 7 simulated ages. Colors indicate different simulated ages, while symbols indicate different stressed volumes. Several points can have the same color and symbol, when they correspond to different heart rates. The healthy young reference case is indicated by a black diamond.

Predicting stroke volume (*SV*) based on the BCG signal has been one of the main objectives since the early days of this technique (Douglas et al., 1913). This section aims at comparing the results of 4 different formulas (labeled *SV*_1_ to *SV*_4_, and described hereafter) to assess stroke volume or stroke volume changes in the dataset of simulated conditions (results obtained when increasing peripheral resistance up to three times its initial value are also available in Section 7 of the Supplementary Material). Figure 10 presents the different scatter plots and the regression lines obtained with the 4 formulas by comparing the stroke volumes estimated with these formulas to their actual values. It also displays the equations of the regression lines and the correlation coefficients for the global correlation analysis, as well as the means of the regression coefficients and the coefficient of variability of the slope (*cv*_slope_) obtained when running 7 different regression analyses for the 7 simulated ages. These results aim at assessing the potential of each formula to allow intra-subject monitoring.

In 1933 already, Abramson suggested a formula to compute the stroke volume, based on BCG recordings and a list of physiological considerations (Abramson, 1933). Starr and colleagues then suggested their own formulas, relying on the amplitudes of the I and/or J waves or, alternatively, on the areas under the I and/or J waves (Starr et al., 1939). Among these formulas, the most accurate appeared to be the following one:


(34) $$S\,V_{1}=\kappa_{S\,V\,1}\,\sqrt{\left(2\,\int I+\int J\right)\,A_{0,\text{Ao}}\,\sqrt{T_{R\,R}}}$$

with *SV*_1_ in ml, κ_*SV*1_ a proportionality factor of 33 cm^2^.mm^–1/2^.s^1/4^ given by Starr, ∫*I* and∫*J* the areas under the I and J waves (in mm.s^–1^), respectively, *A*_*0,Ao*_ in cm^2^, and *T*_*RR*_ in s.

This equation was successfully tested against measurements of stroke volume with the ethyl iodide (Starr et al., 1939) and the Fick (Cournand et al., 1942) methods. Here, Figure 10A presents the comparison of the stroke volumes predicted by Starr’s formula with their actual values (i.e., the values of the stroke volume generated by our computational model). A positive correlation is indeed found when using Equation 34 (*R*^2^ = 0.50), but its predictions overestimate stroke volume. This could be explained by the fact that the low pass filter at 25 Hz underestimates the loss of amplitude due to natural body dampening. When analyzing separately the different simulated ages, the correlation coefficient remains quite low (mean *R*^2^ = 0.60) and the coefficient of variation of the slope is high (0.29). This means that this formula is not ideal to estimate intra-subject variations of stroke volume.

Very recently, some authors still argued that the amplitude of the J wave could be an indicator of relative changes in aortic pulse pressure, which are often well correlated to changes in stroke volume (Kim et al., 2016). A positive correlation between *T*_*RR*_ and the amplitude of the J wave has also been observed (Inan et al., 2009b), indicating that a longer ventricular filling time, and thus a larger stroke volume, have a positive effect on this parameter. The literature is less conclusive about the use of the I wave for a correlation with the stroke volume and Figure 6 shows that it is much more impacted by filtering than the other waves of the longitudinal ***BCG*_**acc**_** signal. As already described and shown in Figure 8B, the I wave is also more affected by aging than the other systolic waves. However, the stroke volume changes caused by aging are too small to explain such a large decrease in the area under the I wave, even when correcting for different aortic cross-section areas. To account for these observations, we adapt the formula suggested by Starr, keeping only the contribution of the J wave. We also choose to optimize the associated proportionality coefficient κ_*SV*2_ to give the best possible estimates based on low pass filtered data at 25 Hz:


(35) $$S\,V_{2}=\kappa_{S\,V\,2}\,\sqrt{\int J\,A_{0,\text{Ao}}\,\sqrt{T_{R\,R}}}$$

with κ_*SV*2_ a proportionality factor of 35 cm^2^.mm^–1/2^.s^1/4^, while ∫*J*, *A*_*0,Ao*_, and *T*_*RR*_ are as in Equation 34.

Figure 10B shows that the correlation coefficient obtained with this formula is better (*R*^2^ = 0.78) than with the initial formula. The scatter of values estimated for a subject at a given age is also much reduced, which is confirmed by the analysis by age (mean *R*^2^ = 0.93). In addition, we find that the coefficient of variation of the slope is very low (*cv*_slope_ = 0.09), which is a very encouraging sign that the formula based only on the area under the J wave would be efficient in reliably assessing intra-subject differences. Judging from the relatively large overestimations observed with Starr’s formula in Figure 10A, we can reasonably assume that the proportionality coefficient should be higher with experimental data. This is also true for the other results presented hereafter.

The use of the integral of ***BCG*_**acc**_** waves in the two formulas suggested above is not ideal. Starr himself admitted that it would make more physical sense to integrate twice the acceleration signal to compute parameters that could correlate to the stroke volume (Starr, 1955). The integral of *BCG*_kin_ has the potential to be such a parameter, since *BCG*_kin_ is computed based on the velocity signal, which is already the integral of ***BCG*_**acc**_**. Recent trials have indeed shown that changes of the integral of *BCG*_kin_ were correlated to changes of stroke volume both in increased (Hossein et al., 2019) and decreased (Rabineau et al., 2020) contractility settings.

Knowing the fact that the aorta is the largest contributor to ***BCG*_**vel**_**, and thus to *BCG*_kin_, a scaling law is established based on *iK*_sys_, the integral of *BCG*_kin_ over the systole (see Section 7 of the Supplementary Material), and leads to:


(36) $$S\,V_{3}=\kappa_{S\,V\,3}\,{\left(\frac{i\,K_{s\,y\,s}\,A_{0,\text{Ao}}^{2}\,L\,V\,E\,T\,W_{b}}{\rho^{2}}\right)}^{1/4}$$

with κ_*SV*3_ a proportionality factor of 14 500 cm^2^.m^–5/4^ optimized based on the results of the simulations, *iK*_*sys*_ in J.s, *A*_*0,Ao*_ in cm^2^, *LVET* in s, *W_b_* in kg, and ρ in kg.m^–3^.

As shown in Figure 10C, a positive correlation is clearly visible, even though the correlation coefficient is relatively low (*R*^2^ = 0.38). However, this formula appears to be efficient in terms of intra-subject monitoring (mean *R*^2^ = 0.82, *cv*_slope_ = 0.11).

To explore the relationship between BCG parameters and stroke volume, we also adapt the second formula (Equation 35) to replace $\sqrt{T_{R\,R}}$ by *LVET* and use the parameter *iK*_*sys*_ instead of ∫*J*:


(37) $$S\,V_{4}=\kappa_{S\,V\,4}\,\sqrt{i\,K_{s\,y\,s}\,A_{0,\text{Ao}}\,L\,V\,E\,T}$$

with κ_*SV*4_ a proportionality factor of 37 300 cm^2^.m^1/2^.kg^–1/2^ optimized based on the results of the simulations, and the other parameters as in Equation 36.

Indeed, *iK*_*sys*_ includes information from all the axes and has thus the potential to be less sensitive than ∫*J* to inter-subject differences in terms of orientation of the heart and the aorta, as well as experimental errors regarding the placement of the BCG device. Beside this, as hinted by the effect of filtering on ***BCG*_**vel**_** and ***BCG*_**acc**_** in Figure 6, *iK*_*sys*_ should be less sensitive to differences in body dampening characteristics, but also to noise. This assumption is evaluated in Section 7 of the Supplementary Material, based on the comparison of estimates extracted from the different filtered signals.

The quality of this fourth formula to correctly assess stroke volume is visible in Figure 10D, where the global correlation coefficient (*R*^2^ = 0.74) is approximately as good as for *SV*_2_, while the mean R^2^ and *cv*_slope_ are similar to those of *SV*_3_.

As in Section “Pulse Wave Velocity,” the different formulas all rely on the knowledge, or the correct estimation, of *A*_*0,Ao*_. If such an estimation is not possible, these formulas can still be used to assess intra-subject changes. Indeed, Figure 10 shows that *SV*_2_, *SV*_3_, and *SV*_4_ are all performing this task better than *SV*_1_, the estimate based on the formula initially suggested by Starr. They have the potential to be used both in the context of longitudinal studies and beat by beat stroke volume assessments. However, prior to this, it is obviously still necessary to gather experimental clues supporting these findings and to define which of these formulas is the most accurate in the clinical practice. Indeed, it is also important to remind that only the formula giving *SV*_1_ has been validated against clinical data so far (Starr et al., 1939).

### Model Limitations

Several possible limitations require consideration and the first of them are linked to assumptions in the model of blood flow used in this study. For instance, we assume a constant value of the ζ_*i*_ parameter defining the velocity profile in each artery, whereas it is normally changing through the cardiac cycle and along the length of each artery. Furthermore, the velocity profile is assumed to show axial symmetry, while it is not the case in some vessels of large curvature like the aortic arch (Seed and Wood, 1971). In this case, the influence of the curvature of the vessels is neglected and we do not consider helicity of the flow, whereas such a feature can sometimes be observed on MRI images, especially in the aorta (Bogren and Buonocore, 1999). We also neglect the deformations of the arteries in the longitudinal direction, which should have a negligible effect on our parameters of interest, even though these deformations may be useful to take into account in some particular cases (Pagoulatou et al., 2018).

Some compartments that are considered as 0D elements would benefit from a 1D representation instead. It is the case for instance in the cerebral arterial tree, since some of these arteries are necessary to ensure a correct representation of blood flow in the proximal cerebral vessels (Reymond et al., 2009). However, the results show that these arteries do not have a large impact on the BCG signal. Still, the ***BCG*_**acc**_** signal generated by the model has some morphological differences with experimental records, such as the absence of H waves in the longitudinal axis. As discussed, in this paper and in the literature (Nickerson, 1949; De Lalla et al., 1950), the H wave is very likely due to blood flow in the vena cavae following the right atrial contraction. Therefore, our model would benefit from the addition of 1D elements in the large veins of the systemic circulation, as already experimented in previous studies (Müller and Toro, 2014; Mynard and Smolich, 2015).

This study focuses on the sole contribution of blood flow to BCG. However, even if the simulated signals presented in this research match experimental observations, the movements of the myocardium may play a non-negligible role in the generation of a BCG signal. More precisely, it is assumed in the model that the position of the center of mass of each cardiac chamber is constant and equal to the position measured on an MR angiography set of images, which may have overestimated the momentum associated to blood flow between cardiac chambers. On the other hand, the absence of myocardium movements may have led to an underestimation of the contribution of the heart to the global BCG signal.

Here we consider only the BCG signal generated by cardiovascular activity in one subject and a given geometry of the arterial tree. In particular, no age-related elongation of the aorta is considered. Even though the hemodynamic impact of this elongation is relatively small (Whittle and Diaz-Artiles, 2021), it can change the orientation of the aorta and affect the BCG signal, including the timings between the different peaks of ***BCG*_**acc**_**. In addition, experimental records show that abnormal shapes of the BCG signal are more and more common with increasing age (Scarborough et al., 1953) and that the proposed scaling laws may not work for such records (Starr, 1955).

The impact of respiration on hemodynamics and BCG is not included in this model. However, the position and orientation of the heart may vary as a function of the breathing phase and have an impact on the BCG signal and its distribution on the different axes (Prisk et al., 2001). The addition of baroreflex mediated arterial resistance and a better description of the venous model would also help better understand the cardiorespiratory interaction using such a computational model.

Finally, even though the effect of low-pass filtering has been evaluated on the BCG signals generated by the computational model, a more realistic bio-mechanical representation of the human body may help better understand the mechanical filtering of the vibratory signals between their sources and the BCG sensors. A simplified model, including separate elements for the upper torso, upper limbs, internal organs, and lower limbs, connected by dampers and springs, has already been studied by Yousefian et al. (2019).

Nonetheless, we believe that these limitations simply point toward future elements to be studied and do not preclude any fundamental conclusion of this research.

## Conclusion

We presented a multiscale computational model of blood flow based on anatomical data obtained by whole-body MR angiography on a healthy young subject. To our knowledge, it is the first time that a model of such a complexity, based on reliable geometric references, is applied to the generation of a 3D artificial BCG signal. In addition, this model can easily be adapted to a variety of simulated cases. Here, we evaluate the impact of aging and apply this model to the computation of artificial multidimensional BCG signals in simulated subjects between 20 and 80 years old, at three different heart rates and three different stressed volumes.

The results show the relative contributions of different cardiovascular elements to the genesis of the BCG signal expressed in different forms. Even if the aorta is the main contributor to the BCG signal, it is not the only one, and a more complete model, like this one, is necessary to generate BCG signals that are close to clinical observations.

On ***BCG*_**acc**_**, the contributions of the *x* and *z* axes to the overall signal are significantly lower than the one of the *y* axis, but they are not negligible. On ***BCG*_**vel**_** the contribution of these axes is even less negligible and they have the potential to bring valuable information about intracardiac blood flows. It remains, however, that the *x* and *z* axes may be strongly affected by other phenomena, including rotations when the subject is not restrained, that require further study.

After low pass filtering with a cutoff frequency at 25 Hz, the shapes and amplitudes of the different axes of ***BCG*_**acc**_** are closer to experimental observations, underlying the dampening effect that the human body may play on high-frequency movements induced by cardiovascular activity. Depending on the application and the type of metrics used, there may be a necessity to correct for different characteristics of dampening caused by different body types.

The shapes and amplitudes of the BCG signal are changing with age and its associated cardiovascular consequences, even when stroke volume is kept relatively constant. The I wave of the longitudinal ***BCG*_**acc**_** is particularly affected by aging and, more generally, the timings and the relative amplitudes of the different waves are changing with age. Here we suggest a scaling law that we apply to several time intervals measured on the longitudinal axis of ***BCG*_**acc**_**, which shows very good correlations with the inverse of the pulse wave velocity. These could prove useful to easily assess relative differences in vascular age between subjects, and even measure the pulse wave velocity.

We also suggested three new formulas to estimate the stroke volume and its changes, based on longitudinal ***BCG*_**acc**_** and *BCG*_kin_. To assess absolute values of the stroke volume, the knowledge of the aortic cross-section area and left ventricular ejection time are necessary. However, intra-subject longitudinal and even beat-by-beat evolution of the stroke volume are still possible even without this information. Beside this, all the three new suggested formulas provide better intra-subject evaluations of stroke volume changes than the formula initially suggested by Starr. To limit the effects that different body types may have on body dampening, and thus estimations of stroke volumes, we suggest using the formulas based on parameters measured on *BCG*_kin_.

In a nutshell, we have shown very encouraging reasons to believe that BCG could help to easily assess some key aspects of the cardiovascular status of a patient. However, all these findings still require confirmation in a clinical setting.

## Nomenclature

*Roman symbols*

**Table:** 

A	Peak ventricular filling flow amplitude during late diastole, m.s^–1^
*A*	Cross-section area of the lumen, m^2^
age	Age of the simulated subject, years
*B*	Flow separation (or Bernoulli) coefficient, mmHg.s^2^.ml^–2^
BCG	Ballistocardiography or ballistocardiogram, depending on the context
*c*	Pulse wave velocity, m.s^–1^
*C*	Elastic wall compliance, ml.mmHg^–1^
*cv*_slope_	Coefficient of variability of the slope in the regression analysis, –
E	Peak ventricular filling flow amplitude during early diastole, m.s^–1^
*E*	Elastance, ml.mmHg^–1^
*e*	Normalized time-varying function related to elastance, –
ECG	Electrocardiography
*f*	Friction force per unit length, kg.s^–2^
***G***	Position vector of a compartment in the reference frame of the body, m
*h*	Arterial wall thickness, m
H	Pre-ejection upward wave of the longitudinal acceleration BCG signal
*H*_*b*_	Body height of the subject, m
I	First downward wave of the longitudinal acceleration BCG signal
’I	First downward wave of the longitudinal velocity BCG signal
*iK*	Integral of the BCG kinetic energy over a given duration, J.s
J	First post-ejection upward wave of the longitudinal acceleration BCG signal
’J	First upward wave of the longitudinal velocity BCG signal
K	Second downward wave of the longitudinal acceleration BCG signal
*K*	Rate coefficient for valve opening or closing, mmHg^–1^.s^–1^
*l*	Arterial length, m
*L*	Fluid inertia coefficient, mmHg.s^2^.ml^–1^
LA	Left atrium
LV	Left ventricle
LVET	Left ventricular ejection time, s
MR	Magnetic resonance
*N*	Total number of compartments
*P*	Pressure, mmHg
$\tilde{P\,T\,T_{△\,t}}$	Parameter meant to approach a pulse transit time based on the time-interval △*t*, s^1/4^.cm^–1^
*Q*	Blood flow rate, ml.s^–1^
*r*	Radial coordinate in a blood vessel, m
R	ECG wave corresponding to depolarization of the main mass of the ventricles
R^2^	Correlation coefficient
*R*	Viscous resistance to the flow, mmHg.s.ml^−1^
ℛ	Radius of the lumen, m
RA	Right atrium
RV	Right ventricle
*s*	Axial coordinate in a blood vessel, oriented in the direction distal to the heart, m
*SV*	Stroke volume, ml
*t*	Time, s
*T*	Duration, s
*u*	Longitudinal blood velocity in a vessel, m.s^–1^
*U*	Cross-section average of longitudinal blood velocity in a vessel, m.s^–1^
$\bar{U}$	Time average of *U*, m.s^–1^
*V*	Volume, ml
*V*_dead_	Dead (or zero-pressure) volume in a cardiac chamber, ml
*W*_*b*_	Body mass of the subject, kg
*x*	Transverse (left to right) component in the reference frame of the body
*y*	Longitudinal (caudo-cranial) component in the reference frame of the body
*z*	Anteroposterior (dorsoventral) component in the reference frame of the body

*Greek symbols*

**Table:** 

α	Momentum-flux correction (or Coriolis) coefficient of the blood velocity profile, –
β	Parameter related to the elasticity of the arterial wall, m.mmHg
γ	Asymmetry ratio in the fractal representation of the pulmonary arterial tree, –
Γ	Parameter related to the viscosity of the arterial wall, m.s.mmHg
Δ	Difference through an interface (P) or short interval (s)
ζ	Constant defining the velocity profile in an artery, –
η	Valve state, –
κ	Proportionality factor used in the scaling laws, units depending on the scaling law
μ	Dynamic viscosity of blood, kg.m^–1^.s^–1^
ξ	Fractal exponent in the fractal representation of the pulmonary arterial tree, –
ρ	Volumetric mass density of blood, kg.m^–3^

*Subscripts*

**Table:** 

*0*	At *P* = *P*_*ref*_
*a*	Atrium
*A*	Amplitude of variation
acc	Acceleration
Ao	Aorta
*B*	Baseline
*CC*	Cardiac chamber
*cp*	Contraction phase
*CoM*	Center of mass
*CV*	Cardiac valve
*d*	Daughter
dist	Distal
eff	Effective
elast	Elastic component (of blood pressure)
ext	Exterior
*i*	Artery number
in	Input
*j*	Compartment number
*k*	Index related to the RLC compartments of the peripheral circulation
kin	Kinetic energy
*m*	Mother
max	Maximal
out	Output
*P*	Poiseuille flow
per	Peripheral
pos	Position
prox	Proximal
ref	Reference
*rp*	Relaxation phase
RR	Interval between two R waves, s
sys	Systole
up	Compartment immediately upstream (assuming physiological flow of blood)
*v*	Ventricle
*vc*	Valve closing
vel	Velocity
visc	Viscous component (of blood pressure)
*vo*	Valve opening

*Superscripts*

**Table:** 

25*y*	Reference case for a subject aged 25.

## Data Availability Statement

The original contributions presented in the study are included in the article/Supplementary Material, further inquiries can be directed to the corresponding author/s.

## Author Contributions

P-FM initiated the idea of this project. JR and BH designed the computational model. TL provided whole-body MR angiography data. JR performed the different simulations with the computational model and takes responsibility for the integrity of the data and the accuracy of data analysis, and drafted the manuscript. BH, AN, TL, PB, and P-FM critically revised the manuscript for important intellectual content. All authors did proofreading and corrections for this manuscript.

## Conflict of Interest

P-FM is co-founder and hold shares of HeartKinetics, a company specialized in cardiac monitoring. The remaining authors declare that the research was conducted in the absence of any commercial or financial relationships that could be construed as a potential conflict of interest.

## Publisher’s Note

All claims expressed in this article are solely those of the authors and do not necessarily represent those of their affiliated organizations, or those of the publisher, the editors and the reviewers. Any product that may be evaluated in this article, or claim that may be made by its manufacturer, is not guaranteed or endorsed by the publisher.

## References

[B1] Abramson E. (1933). Die Rückstoßkurve des Herzens (Kardiodynamogramm). *Skand. Arch. Für Physiol.* 66 191–224. 10.1111/j.1748-1716.1933.tb01031.x

[B2] Alastruey J. Parker K. H. Peiro J. Sherwin S. J. (2008). Lumped parameter outflow models for 1-D blood flow simulations: effect on pulse waves and parameter estimation. *Commun. Comput. Phys.* 4 317–336.

[B3] Alastruey J. Parker K. H. Sherwin S. J. (2012). “Arterial pulse wave haemodynamics,” in *Proceedings of the 11th International Conference on Pressure Surges*, ed. Anderson S. (Bedford: British Hydromechanics Research (BHR) Group), 401–443.

[B4] Auslander D. M. Lobdell T. E. Chong D. (1972). A large-scale model of the human cardiovascular system and its application to ballistocardiography. *J. Dyn. Syst. Meas. Control* 94 230–238. 10.1115/1.3426593 4750731

[B5] Aydemir V. B. Nagesh S. Shandhi M. H. Fan J. Klein L. Etemadi M. (2020). Classification of decompensated heart failure from clinical and home ballistocardiography. *IEEE Trans. Biomed. Eng.* 67 1303–1313. 10.1109/TBME.2019.2935619 31425011 PMC7271768

[B6] Bogren H. G. Buonocore M. H. (1999). 4D magnetic resonance velocity mapping of blood flow patterns in the aorta in young vs. elderly normal subjects. *J. Magn. Reson. Imaging* 10 861–869. 10.1002/(SICI)1522-2586(199911)10:5<861::AID-JMRI35<3.0.CO;2-E 10548800

[B7] Brown H. R. Hoffman M. J. De Lalla V. (1950). Ballistocardiographic findings in patients with symptoms of angina pectoris. *Circulation* 1 132–140. 10.1161/01.CIR.1.1.132 15401201

[B8] Buess A. Van Muylem A. Nonclercq A. Haut B. (2020). Modeling of the transport and exchange of a gas species in lungs with an asymmetric branching pattern. Application to nitric oxide. *Front. Physiol.* 11:570015. 10.3389/fphys.2020.570015 33362572 PMC7758446

[B9] Caro C. G. Pedley T. J. Schroter R. C. Seed W. A. (eds). (2012). *The Mechanics of the Circulation*, 2nd Edn. Cambridge: Cambridge University Press.

[B10] Charlton P. H. Mariscal Harana J. Vennin S. Li Y. Chowienczyk P. Alastruey J. (2019). Modeling arterial pulse waves in healthy aging: a database for in silico evaluation of hemodynamics and pulse wave indexes. *Am. J. Physiol. Heart Circ. Physiol.* 317 H1062–H1085. 10.1152/ajpheart.00218.2019 31442381 PMC6879924

[B11] Chen C.-H. Nakayama M. Nevo E. Fetics B. J. Maughan W. L. Kass D. A. (1998). Coupled systolic-ventricular and vascular stiffening with age: implications for pressure regulation and cardiac reserve in the elderly. *J. Am. Coll. Cardiol.* 32 1221–1227. 10.1016/S0735-1097(98)00374-X 9809929

[B12] Cournand A. Ranges H. A. Riley R. L. (1942). Comparison of results of the normal ballistocardiogram and a direct Fick method in measuring the cardiac output in man. *J. Clin. Invest.* 21 287–294. 10.1172/JCI101302 16694914 PMC435142

[B13] De Lalla V. Epstein M. A. Brown H. R. (1950). Analysis of H wave of ballistocardiogram. *Circulation* 2 765–769. 10.1161/01.CIR.2.5.765 14783830

[B14] Dietenbeck T. Houriez-Gombaud-Saintonge S. Charpentier E. Gencer U. Giron A. Gallo A. (2021). Quantitative magnetic resonance imaging measures of three-dimensional aortic morphology in healthy aging and hypertension. *J. Magn. Reson. Imaging* 53 1471–1483. 10.1002/jmri.27502 33426700

[B15] Douglas C. G. Haldane J. S. Henderson Y. Schneider E. C. (1913). VI. Physiological observations made on Pike’s Peak, Colorado, with special reference to adaptation to low barometric pressures. *Philos. Trans. R. Soc. Lond. Ser. B Contain. Pap. Biol. Character* 203 185–318. 10.1098/rstb.1913.0006

[B16] Etemadi M. Inan O. T. (2018). Wearable ballistocardiogram and seismocardiogram systems for health and performance. *J. Appl. Physiol.* 124 452–461. 10.1152/japplphysiol.00298.2017 28798198 PMC5867366

[B17] Felderhoff B. Klensch H. (1960). Die altersbedingte Formwandlung des ultraniederfrequenten Ballistokardiogramms. *Pflüg. Arch. Für Gesamte Physiol. Menschen Tiere* 272 13–14. 10.1007/BF00680916

[B18] Formaggia L. Lamponi D. Tuveri M. Veneziani A. (2006). Numerical modeling of 1D arterial networks coupled with a lumped parameters description of the heart. *Comput. Methods Biomech. Biomed. Engin.* 9 273–288. 10.1080/10255840600857767 17132614

[B19] Gallo C. Ridolfi L. Scarsoglio S. (2020). “A closed-loop multiscale model of the cardiovascular system: application to heart pacing and open-loop response,” in *Proceedings of the XV Mediterranean Conference on Medical and Biological Engineering and Computing – MEDICON 2019 IFMBE*, eds Henriques J. Neves N. de Carvalho P. (Cham: Springer International Publishing), 577–585. 10.1007/978-3-030-31635-8_69

[B20] Giovangrandi L. Inan O. T. Wiard R. M. Etemadi M. Kovacs G. T. A. (2011). Ballistocardiography – a method worth revisiting. *Annu. Int. Conf. IEEE Eng. Med. Biol. Soc.* 2011 4279–4282. 10.1109/IEMBS.2011.6091062 22255285 PMC4274997

[B21] Gómez-Clapers J. Serra Rocamora A. Casanella Alonso R. Pallàs Areny R. (2013). *Uncertainty Factors in Time-Interval Measurements in Ballistocardiography.* 395–399. Available online at: https://upcommons.upc.edu/handle/2117/21545 (accessed March 30, 2021).

[B22] Gordon J. W. (1877). Certain molar movements of the human body produced by the circulation of the blood. *J. Anat. Physiol.* 11 533–536. PMC1309740 17231163

[B23] Guidoboni G. Sala L. Enayati M. Sacco R. Szopos M. Keller J. (2017). Cardiovascular function and ballistocardiogram: a relationship interpreted via mathematical modeling. *IEEE Trans. Biomed. Eng.* 66 2906–2917. 10.1109/TBME.2019.2897952 30735985 PMC6752973

[B24] Hickson S. S. Butlin M. Graves M. Taviani V. Avolio A. P. McEniery C. M. (2010). The relationship of age with regional aortic stiffness and diameter. *JACC Cardiovasc. Imaging* 3 1247–1255. 10.1016/j.jcmg.2010.09.016 21163453

[B25] Hixson W. C. Beischer D. E. (1964). *Biotelemetry of the Triaxial Ballistocardiogram and Electrocardiogram in a Weightless Environment.* Pensacola, FL: Naval School of Aviation Medicine. 14292679

[B26] Horeman H. W. Noordergraaf A. (1958). Numerical evaluation of volume pulsations in man I. The basic formula. *Phys. Med. Biol.* 3 51–58. 10.1088/0031-9155/3/1/307 13578698

[B27] Hossein A. Mirica D. C. Rabineau J. Rio J. I. D. Morra S. Gorlier D. (2019). Accurate detection of dobutamine-induced haemodynamic changes by kino-cardiography: a randomised double-blind placebo-controlled validation study. *Sci. Rep.* 9:10479. 10.1038/s41598-019-46823-3 31324831 PMC6642180

[B28] Hossein A. Rabineau J. Gorlier D. Del Rio J. I. J. van de Borne P. Migeotte P.-F. (2021a). Kinocardiography derived from ballistocardiography and seismocardiography shows high repeatability in healthy subjects. *Sensors* 21:815. 10.3390/s21030815 33530417 PMC7865512

[B29] Hossein A. Rabineau J. Gorlier D. Pinki F. van de Borne P. Nonclercq A. (2021b). Effects of acquisition device, sampling rate, and record length on kinocardiography during position-induced haemodynamic changes. *Biomed. Eng. Online* 20:3. 10.1186/s12938-020-00837-5 33407507 PMC7788803

[B30] Hudsmith L. Petersen S. Francis J. Robson M. Neubauer S. (2005). Normal human left and right ventricular and left atrial dimensions using steady state free precession magnetic resonance imaging. *J. Cardiovasc. Magn. Reson.* 7 775–782. 10.1080/10976640500295516 16353438

[B31] Hughes T. J. R. Lubliner J. (1973). On the one-dimensional theory of blood flow in the larger vessels. *Math. Biosci.* 18 161–170. 10.1016/0025-5564(73)90027-8

[B32] Inan O. T. Etemadi M. Paloma A. Giovangrandi L. Kovacs G. T. A. (2009a). Non-invasive cardiac output trending during exercise recovery on a bathroom-scale-based ballistocardiograph. *Physiol. Meas.* 30 261–274. 10.1088/0967-3334/30/3/003 19202234

[B33] Inan O. T. Etemadi M. Wiard R. M. Giovangrandi L. Kovacs G. T. A. (2009b). Robust ballistocardiogram acquisition for home monitoring. *Physiol. Meas.* 30 169–185. 10.1088/0967-3334/30/2/005 19147897

[B34] Inan O. T. Migeotte P.-F. Park K.-S. Etemadi M. Tavakolian K. Casanella R. (2015). Ballistocardiography and seismocardiography: a review of recent advances. *IEEE J. Biomed. Health Inform.* 19 1414–1427. 10.1109/JBHI.2014.2361732 25312966

[B35] Kim C.-S. Ober S. L. McMurtry M. S. Finegan B. A. Inan O. T. Mukkamala R. (2016). Ballistocardiogram: mechanism and potential for unobtrusive cardiovascular health monitoring. *Sci. Rep.* 6:31297. 10.1038/srep31297 27503664 PMC4977514

[B36] Klabunde R. (2011). *Cardiovascular Physiology Concepts.* Philadelphia, PA: Lippincott Williams & Wilkins.

[B37] Knoop A. A. (1965). *Experimental Investigations on Ultra-low Frequency Displacement Ballistocardiography.* Washington, DC: National Aeronautics and Space Administration. 14292663

[B38] Lakatta E. G. Gerstenblith G. Angell C. S. Shock N. W. Weisfeldt M. L. (1975). Prolonged contraction duration in aged myocardium. *J. Clin. Invest.* 55 61–68. 10.1172/JCI107918 1109181 PMC301717

[B39] Liang F. Takagi S. Himeno R. Liu H. (2009a). Multi-scale modeling of the human cardiovascular system with applications to aortic valvular and arterial stenoses. *Med. Biol. Eng. Comput.* 47 743–755. 10.1007/s11517-009-0449-9 19198911

[B40] Liang F. Takagi S. Himeno R. Liu H. (2009b). Biomechanical characterization of ventricular–arterial coupling during aging: a multi-scale model study. *J. Biomech.* 42 692–704. 10.1016/j.jbiomech.2009.01.010 19261285

[B41] Magder S. (2016). Volume and its relationship to cardiac output and venous return. *Crit. Care* 20:271. 10.1186/s13054-016-1438-7 27613307 PMC5018186

[B42] Marcus F. I. Sorrell V. Zanetti J. Bosnos M. Baweja G. Perlick D. (2007). Accelerometer-derived time intervals during various pacing modes in patients with biventricular pacemakers: comparison with normals. *Pacing Clin. Electrophysiol.* 30 1476–1481. 10.1111/j.1540-8159.2007.00894.x 18070301

[B43] McLeod J. (1964). Computer simulation of the hydrodynamics of the cardiovascular system. *Simulation* 2 33–41. 10.1177/003754976400200311

[B44] Migeotte P.-F. Mucci V. Delière Q. Lejeune L. van de Borne P. (2016). “Multi-dimensional kineticardiography a new approach for wearable cardiac monitoring through body acceleration recordings,” in *Proceedings of the XIV Mediterranean Conference on Medical and Biological Engineering and Computing 2016*, eds Kyriacou E. Christofides S. Pattichis C. S. (Cham: Springer International Publishing), 1125–1130. 10.1007/978-3-319-32703-7_220

[B45] Migeotte P.-F. Tank J. Pattyn N. Funtova I. Baevsky R. Neyt X. (2011). “Three dimensional ballistocardiography: methodology and results from microgravity and dry immersion,” in *Peoceedings of the 2011 Annual International Conference of the IEEE Engineering in Medicine and Biology Society* (Boston, MA), 4271–4274. 10.1109/IEMBS.2011.6091060 22255283

[B46] Morra S. Hossein A. Gorlier D. Rabineau J. Chaumont M. Migeotte P.-F. (2020). Ballistocardiography and seismocardiography detection of hemodynamic changes during simulated obstructive apnea. *Physiol. Meas.* 41:065007. 10.1088/1361-6579/ab924b 32396890

[B47] Müller L. O. Toro E. F. (2014). A global multiscale mathematical model for the human circulation with emphasis on the venous system. *Int. J. Numer. Methods Biomed. Eng.* 30 681–725. 10.1002/cnm.2622 24431098

[B48] Murgo J. P. Westerhof N. Giolma J. P. Altobelli S. A. (1980). Aortic input impedance in normal man: relationship to pressure wave forms. *Circulation* 62 105–116. 10.1161/01.CIR.62.1.105 7379273

[B49] Mynard J. P. Davidson M. R. Penny D. J. Smolich J. J. (2012). A simple, versatile valve model for use in lumped parameter and one-dimensional cardiovascular models. *Int. J. Numer. Methods Biomed. Eng.* 28 626–641. 10.1002/cnm.1466 25364842

[B50] Mynard J. P. Smolich J. J. (2015). One-dimensional haemodynamic modeling and wave dynamics in the entire adult circulation. *Ann. Biomed. Eng.* 43 1443–1460. 10.1007/s10439-015-1313-8 25832485

[B51] Nadler S. B. Hidalgo J. H. Bloch T. (1962). Prediction of blood volume in normal human adults. *Surgery* 51 224–232. 21936146

[B52] Nakou E. Parthenakis F. Kallergis E. Marketou M. Nakos K. Vardas P. (2016). Healthy aging and myocardium: a complicated process with various effects in cardiac structure and physiology. *Int. J. Cardiol.* 209 167–175. 10.1016/j.ijcard.2016.02.039 26896615

[B53] Nichols W. W. O’Rourke M. F. Vlachopoulos C. (eds). (2011). *McDonald’s Blood Flow in Arteries: Theoretic, Experimental, and Clinical Principles*, 6th Edn. London: Hodder Arnold.

[B54] Nickerson J. L. (1949). Some observations on the ballistocardiographic pattern, with special reference to the H and K waves. *J. Clin. Invest.* 28 369–377. 10.1172/JCI102079 16695686 PMC439610

[B55] Nikitin N. P. Witte K. K. A. Ingle L. Clark A. L. Farnsworth T. A. Cleland J. G. F. (2005). Longitudinal myocardial dysfunction in healthy older subjects as a manifestation of cardiac ageing. *Age Ageing* 34 343–349. 10.1093/ageing/afi043 15734747

[B56] Noordergraaf A. (1967). Ballistocardiography: the ballistocardiogram as a source of hæmodynamic information. *Proc. R. Soc. Med.* 60 1286–1289. 10.1177/003591576706001220 6066578 PMC1901491

[B57] Noordergraaf A. Horeman H. W. (1958). Numerical evaluation of volume pulsations in man II. Calculated volume pulsations of forearm and calf. *Phys. Med. Biol.* 3 59–70. 10.1088/0031-9155/3/1/308 13578699

[B58] Noordergraaf A. Horeman H. W. Holt S. P. T. van Dongen R. (1959). Numerical evaluation of volume pulsations in man IV. The calculation of the human ballistocardiogram. *Phys. Med. Biol.* 3 349–360. 10.1088/0031-9155/3/4/304 13674896

[B59] Noordergraaf A. Verdouw P. D. Boom H. B. K. (1963). The use of an analog computer in a circulation model. *Prog. Cardiovasc. Dis.* 5 419–439. 10.1016/S0033-0620(63)80009-2 13938863

[B60] Pagoulatou S. Ferraro M. Trachet B. Bikia V. Adamopoulos D. Stergiopulos N. (2018). P46 elongation of the proximal aorta during the cardiac cycle plays an important role in the estimation of aortic compliance. *Artery Res.* 24 91–91. 10.1016/j.artres.2018.10.099

[B61] Pedley T. J. (ed.) (1980). *The Fluid Mechanics of Large Blood Vessels.* Cambridge: Cambridge University Press.

[B62] Peverill R. E. (2019). Aging and the relationships between long-axis systolic and early diastolic excursion, isovolumic relaxation time and left ventricular length—Implications for the interpretation of aging effects on e‘. *PLoS One* 14:e0210277. 10.1371/journal.pone.0210277 30615676 PMC6322720

[B63] Prisk G. K. Verhaeghe S. Padeken D. Hamacher H. Paiva M. (2001). Three-dimensional ballistocardiography and respiratory motion in sustained microgravity. *Aviat. Space Environ. Med.* 72 1067–1074. 11763106

[B64] Quarteroni A. Tuveri M. Veneziani A. (2000). Computational vascular fluid dynamics: problems, models and methods. *Comput. Vis. Sci.* 2 163–197. 10.1007/s007910050039

[B65] Qureshi M. U. Vaughan G. D. A. Sainsbury C. Johnson M. Peskin C. S. Olufsen M. S. (2014). Numerical simulation of blood flow and pressure drop in the pulmonary arterial and venous circulation. *Biomech. Model. Mechanobiol.* 13 1137–1154. 10.1007/s10237-014-0563-y 24610385 PMC4183203

[B66] Rabineau J. Hossein A. Landreani F. Haut B. Mulder E. Luchitskaya E. (2020). Cardiovascular adaptation to simulated microgravity and countermeasure efficacy assessed by ballistocardiography and seismocardiography. *Sci. Rep.* 10:17694. 10.1038/s41598-020-74150-5 33077727 PMC7573608

[B67] Redfield M. M. Jacobsen S. J. Borlaug B. A. Rodeheffer R. J. Kass D. A. (2005). Age- and gender-related ventricular-vascular stiffening. *Circulation* 112 2254–2262. 10.1161/CIRCULATIONAHA.105.541078 16203909

[B68] Reymond P. Merenda F. Perren F. Rüfenacht D. Stergiopulos N. (2009). Validation of a one-dimensional model of the systemic arterial tree. *Am. J. Physiol. Heart Circ. Physiol.* 297 H208–H222. 10.1152/ajpheart.00037.2009 19429832

[B69] Scarborough W. R. Davis F. W. Baker B. M. Mason R. E. Singewald M. L. Lore S. A. (1953). A ballistocardiographic study of 369 apparently normal persons: an analysis of “normal” and “borderline” ballistocardiograms. *Am. Heart J.* 45 161–189. 10.1016/0002-8703(53)90178-1 13016476

[B70] Scarborough W. R. Talbot S. A. Braunstein J. R. Rappaport M. B. Dock W. Scarborough W. R. (1956). Proposals for ballistocardiographic nomenclature and conventions: revised and extended. *Circulation* 14 435–450. 10.1161/01.CIR.14.3.435 13365056

[B71] Scarsoglio S. Gallo C. Ridolfi L. (2018). Effects of atrial fibrillation on the arterial fluid dynamics: a modelling perspective. *Meccanica* 53 3251–3267. 10.1007/s11012-018-0867-6

[B72] Seed W. A. Wood N. B. (1971). Velocity patterns in the aorta. *Cardiovasc. Res.* 5 319–330. 10.1093/cvr/5.3.319 5558729

[B73] Senzaki H. Chen C.-H. Kass D. A. (1996). Valvular heart disease/heart failure/hypertension: single-beat estimation of end-systolic pressure-volume relation in humans a new method with the potential for noninvasive application. *Circulation* 94 2497–2506. 8921794 10.1161/01.cir.94.10.2497

[B74] Sherwin S. J. Franke V. Peiró J. Parker K. (2003). One-dimensional modelling of a vascular network in space-time variables. *J. Eng. Math.* 47 217–250. 10.1023/B:ENGI.0000007979.32871.e2

[B75] Smith N. P. Pullan A. J. Hunter P. J. (2002). An anatomically based model of transient coronary blood flow in the heart. *SIAM J. Appl. Math.* 62 990–1018. 10.1137/S0036139999355199

[B76] Soames R. W. Atha J. (1982). Three-dimensional ballistocardiographic responses to changes of posture. *Clin. Phys. Physiol. Meas.* 3 169–177. 10.1088/0143-0815/3/3/001 7140155

[B77] Starr I. (1955). Studies made by simulating systole at necropsy. VI. Estimation of cardiac stroke volume from the ballistocardiogram. *J. Appl. Physiol.* 8 315–329. 10.1152/jappl.1955.8.3.315 13271263

[B78] Starr I. Noordergraaf A. (1967). *Ballistocardiography in Cardiovascular Research–Physical Aspects of the Circulation in Health and Disease.* Amsterdam: North-Holland Publishing Company.

[B79] Starr I. Rawson A. J. (1941). The vertical ballistocardiograph; experiments on the changes in the circulation on arising; with a further study of ballistic theory. *Am. J. Physiol. Leg. Content* 134 403–425. 10.1152/ajplegacy.1941.134.2.403

[B80] Starr I. Rawson A. J. Schroeder H. A. Joseph N. R. (1939). Studies on the estimation of cardiac ouptut in man, and of abnormalities in cardiac function, from the heart’s recoil and the blood’s impacts; the ballistocardiogram. *Am. J. Physiol. Leg. Content* 127 1–28. 10.1152/ajplegacy.1939.127.1.1

[B81] Starr I. Wood F. C. (1961). Twenty-year studies with the ballistocardiograph. *Circulation* 23 714–732. 10.1161/01.CIR.23.5.714

[B82] Strong P. (1970). *Biophysical Measurements*, 1st Edn. Beaverton, OR: Tektronix.

[B83] Suga H. Sagawa K. Shoukat A. A. (1973). Load independence of the instantaneous pressure-volume ratio of the canine left ventricle and effects of epinephrine and heart rate on the ratio. *Circ. Res.* 32 314–322. 10.1161/01.RES.32.3.314 4691336

[B84] Sun Y. Beshara M. Lucariello R. J. Chiaramida S. A. (1997). A comprehensive model for right-left heart interaction under the influence of pericardium and baroreflex. *Am. J. Physiol. Heart Circ. Physiol.* 272 H1499–H1515. 10.1152/ajpheart.1997.272.3.h1499 9087629

[B85] Tannenbaum O. Vesell H. Schack J. A. (1954). The spatial vectorballistocardiogram. *Am. Heart J.* 48 562–572. 10.1016/0002-8703(54)90121-0 13197286

[B86] The Reference Values for Arterial Stiffness’ Collaboration (2010). Determinants of pulse wave velocity in healthy people and in the presence of cardiovascular risk factors: ‘establishing normal and reference values’. *Eur. Heart J.* 31 2338–2350. 10.1093/eurheartj/ehq165 20530030 PMC2948201

[B87] Tortoli P. Bambi G. Guidi F. Muchada R. (2002). Toward a better quantitative measurement of aortic flow. *Ultrasound Med. Biol.* 28 249–257. 10.1016/S0301-5629(01)00462-8 11937288

[B88] van de Vosse F. N. Stergiopulos N. (2011). Pulse wave propagation in the arterial tree. *Annu. Rev. Fluid Mech.* 43 467–499. 10.1146/annurev-fluid-122109-160730

[B89] Villari B. Baselli G. Schneider J. Chiariello M. Hess O. M. (1997). Age Dependency of left ventricular diastolic function in pressure overload hypertrophy. *J. Am. Coll. Cardiol.* 29 181–186. 10.1016/S0735-1097(96)00440-8 8996312

[B90] Westerhof N. Bosman F. De Vries C. J. Noordergraaf A. (1969). Analog studies of the human systemic arterial tree. *J. Biomech.* 2 121–143. 10.1016/0021-9290(69)90024-4 16335097

[B91] Whittle R. S. Diaz-Artiles A. (2021). Modeling individual differences in cardiovascular response to gravitational stress using a sensitivity analysis. *J. Appl. Physiol.* 130 1983–2001. 10.1152/japplphysiol.00727.2020 33914657 PMC8285610

[B92] Wooten S. V. Moestl S. Chilibeck P. Alvero Cruz J. R. Mittag U. Tank J. (2021). Age- and sex-differences in cardiac characteristics determined by echocardiography in masters athletes. *Front. Physiol.* 11:1863. 10.3389/fphys.2020.630148 33536945 PMC7848176

[B93] Yousefian P. Shin S. Mousavi A. S. Kim C.-S. Finegan B. McMurtry M. S. (2019). Physiological association between limb ballistocardiogram and arterial blood pressure waveforms: a mathematical model-based analysis. *Sci. Rep.* 9:5146. 10.1038/s41598-019-41537-y 30914687 PMC6435670

[B94] Zakeri V. Tavakolian K. Blaber A. P. Bauer E. P. Dehkordi P. Khosrow-khavar F. (2020). The repeatability of estimated systolic time intervals in healthy subjects using seismocardiogram and electrocardiogram. *Physiol. Meas.* 41:02NT01. 10.1088/1361-6579/ab6f53 31972547

