# Supplementary material for: Closed-Loop Multiscale Computational Model of Human Blood Circulation. Applications to Ballistocardiography
Source: Front Physiol. 2021 Dec 9;12:734311. doi: 10.3389/fphys.2021.734311 (PMC8697684; doi:10.3389/fphys.2021.734311)
Supplement: Supplementary file 2 [file Data_Sheet_2.pdf]

Supplementary Material

1 Supplementary Data

The geometrical data of the reference healthy young case considered in this study is available as supplementary material. It is based on the segmentation of 55 systemic arteries (see Table 2), 7 pulmonary arteries (see Table 3), and the 4 cardiac chambers, using magnetic resonance angiography data collected on a healthy female (25 years old, 172 cm, 71 kg).

Data is provided as one .csv file per element (artery or cardiac chamber). The first column corresponds to the coordinate (in mm) on the x axis (lateral, oriented from left to right). The second column corresponds to the coordinate (in mm) on the y axis (longitudinal, oriented from the feet to the head). The third column corresponds to the coordinate (in mm) on the z axis (anteroposterior, oriented from the stomach to the back). When applicable, the fourth column corresponds to the radius along the vessel.

2 Normalized time-varying function in the model of cardiac elastance

In Equation 2, defining the time-varying elastance of a cardiac chamber, we use a normalized time varying function $e_{CC}(t)$ defined as in (Liang et al., 2009). Considering that the left and right ventricles (CC = LV and RV, respectively) contract simultaneously at the very beginning of the cardiac cycle, the latter function can be written:

$$e_{CC}(t) = \begin{cases} 0.5\left[1 - \cos\left(\pi t / T_{vcp}\right)\right] & 0 \leq t \leq T_{vcp} \\ 0.5\left[1 + \cos\left(\pi (t - T_{vcp}) / T_{vrp}\right)\right] & T_{vcp} < t \leq T_{vcp} + T_{vrp} \\ 0 & T_{vcp} + T_{vrp} < t \leq T_{RR} \end{cases} \quad \text{(A1)}$$

with T_{RR} the duration of the cardiac cycle, T_{vcp} the duration of the ventricular contraction, and T_{vrp} the duration of the ventricular relaxation. A similar model is chosen for the left and right atria (CC = LA and RA, respectively), which are also assumed to contract simultaneously. However, the time dependence of the corresponding normalized elasticity functions $e_{LA}(t) = e_{RA}(t)$ is changed so that the atrial contraction starts at $0.8\,T_{RR}$ (Gallo et al., 2020). We write T_{acp} the duration of the atrial contraction and T_{arp} the duration of the atrial relaxation. This gives the following equation for the atria:

$$e_{CC}(t) = \begin{cases} 0.5\left[1 + \cos\left(\pi (t - T_{acp} + 0.2\,T_{RR}) / T_{arp}\right)\right] & 0 \leq t \leq T_{acp} + T_{arp} - 0.2\,T_{RR} \\ 0.5\left[1 - \cos\left(\pi (t - 0.8\,T_{RR}) / T_{acp}\right)\right] & 0.8\,T_{RR} < t \leq T_{acp} + 0.8\,T_{RR} \\ 0.5\left[1 + \cos\left(\pi (t - T_{acp} - 0.8\,T_{RR}) / T_{arp}\right)\right] & T_{acp} + 0.8\,T_{RR} < t \leq T_{RR} \\ 0 & \text{otherwise} \end{cases} \quad \text{(A2)}$$

The numerical values used to characterize the time-varying elastance in each cardiac chamber are taken from (Liang et al., 2009) and given in Table 1 (core of the manuscript) for the case of normal contractility and $T_{RR} = 0.86\,s$ (corresponding to a heart rate of 70 bpm).

3 Empirical laws used for the arteries of the healthy young case

In the systemic circulation, an empirical law is used to compute the pulse wave velocity $c_0(s)$ based on the radius $\mathcal{R}(s)$ extracted from the segmentation of the systemic arterial tree:

$$c_0(s) = \sqrt{\frac{2}{3\rho}\left(k_1 e^{k_2\,\mathcal{R}(s)} + k_3\right)} \tag{A3}$$

with the radius $\mathcal{R}(s)$ measured in meters and the coefficients $k_1 = 4.0 \times 10^5\ \mathrm{kg.s^{-2}.m^{-1}}$, $k_2 = -500\ \mathrm{m^{-1}}$, and $k_3 = 40.0 \times 10^3\ \mathrm{kg.s^{-2}.m^{-1}}$ adapted from (Mynard and Smolich, 2015) to achieve normal wave velocities in both the large and smaller vessels (5.08 to $13.58\ \mathrm{m.s^{-1}}$).

We can then compute the coefficient $\beta(s)$, based on the following relationship (Alastruey et al., 2012):

$$\beta(s) = 2\sqrt{\pi}\,\rho\, c_0(s)^2 \mathcal{R}(s) \tag{A4}$$

Another empirical law gives the viscous parameter $\Gamma(s)$ (in m.s.mmHg):

$$\Gamma(\mathrm{s}) = \frac{b_2}{\mathcal{R}(s)} - b_1 \tag{A5}$$

with the coefficient $b_1 = 3.0\ \times 10^{-3}\ \mathrm{m.s.mmHg}$ and $b_2 = 3.8\ \times 10^{-6}\ \mathrm{m^2.s.mmHg}$ adapted from (Mynard and Smolich, 2015).

In the pulmonary circulation, the pulse wave velocity is also assumed to follow Equation A3, with $k_1 = 1.3 \times 10^5\ \mathrm{kg.s^{-2}.m^{-1}}$, $k_2 = -700\ \mathrm{m^{-1}}$, and $k_3 = 12.2 \times 10^3\ \mathrm{kg.s^{-2}.m^{-1}}$. $\beta(s)$ is then computed based on the pulse wave velocity thanks to Equation A4. The viscous parameter $\Gamma(\mathrm{s})$ is supposed to be constant: $\Gamma(\mathrm{s}) = 1.5\ \times 10^{-3}\ \mathrm{m.s.mmHg}$ (Mynard and Smolich, 2015).

4 Correction of the friction force at the wall in the case of non-established flow

In the case of a Poiseuille flow (i.e., a parabolic velocity profile) of an incompressible viscous fluid developed through the entire length of a straight cylindrical artery i, the resistance to the flow is minimal and given by (Pedley et al., 1970a):

$$R_{P,i} = \frac{8\,\mu\, l_i}{\pi\, \mathcal{R}_i^{\,4}} \tag{A6}$$

with l_i and $\mathcal{R}_i$ the length and radius of the artery i, respectively, and μ the dynamic viscosity of blood.

However, it has been shown that the hypothesis of a fully developed Poiseuille flow is not always valid, especially in the large proximal systemic arteries (Seed and Wood, 1971; Bogren and Buonocore, 1999; Tortoli et al., 2002). Hence, it has been suggested to correct the Poiseuille resistance to the flow by multiplying it by a factor Z_i (Pedley et al., 1970a, 1970b) that can be expressed as the ratio of the wall

shear stress in the artery i to the wall shear stress that would take place if the Poiseuille flow was established (see supplementary material in (Buess et al., 2020)):

$$Z_i = \sqrt{\frac{\rho\, \bar{U}_i\, \mathcal{R}_i^{\,2}}{4\, \mu\, l_i}} \tag{A7}$$

with $\bar{U}_i$ the time average of the mean cross-sectional velocity in artery i, and ρ the volumetric mass density of blood.

If the evaluation of Z_i gives a result smaller than 1, it is then set to 1, in agreement with the fact that the Poiseuille resistance corresponds to the minimal resistance to blood flow. This means that we can introduce a critical length of the artery $l_{P,i}$, needed to establish a Poiseuille flow:

$$l_{P,i} = \frac{\rho\ \mathcal{R}_i^{\,2}\, \bar{U}_i}{4\, \mu} \tag{A8}$$

This definition indeed leads to:

$$Z_i = \sqrt{\frac{l_{P,i}}{l_i}} \tag{A9}$$

giving $Z_i > 1$ when $l_i < l_{P,i}$.

We can also express the non-establishment of the flow with the parameters ζ_i and $\alpha_i = \frac{\zeta_i+2}{\zeta_i+1}$ defining the velocity profile in the artery i, as Z_i is the ratio of the actual friction force at the wall per unit length (f, see Equation 11) to its value if the velocity profile was parabolic (i.e., if $\zeta_i = 2$):

$$Z_i = \frac{\zeta_i + 2}{4} \tag{A10}$$

leading to:

$$\zeta_i = 4\sqrt{\frac{l_{P,i}}{l_i}} - 2 \tag{A11}$$

Based on this, an iterative approach is chosen to solve the equations defining blood flow in the arterial tree. First, we assume a Poiseuille flow (i.e., $Z_i = 1$) in all the arteries, which allows a first estimation of blood flow in each artery i and the computation of the associated correction factors Z_i (or, alternatively, α_i Coriolis coefficients). Then, the system of equations is solved a second time, considering the non-establishment of blood flow in some arteries.

5 Comparison with experimental results

The comparison of an artificial 3D BCG signal produced by a numerical model with an experimental 3D BCG signal is a relatively difficult task. Indeed, the signals along the x and z axes are very sensitive to the experimental conditions and, since their amplitudes are usually lower than the one of the y axis, they are easily contaminated by other phenomena (lower signal-to-noise ratios). Depending on the way BCG is recorded, these phenomena can include the precordial movements (contamination by the seismocardiographic signals, especially on the z axis), the movements induced by the rotation of the body (especially on the x axis (Baan et al., 1966)), the movements induced by local flow of blood below the BCG sensor (usually on the z axis), or the movements that happen on the y axis but that are recorded on the x or z axes (depending on the exact position of the subject and of the BCG sensor).

Supplementary Figure 1 displays the 3D acceleration BCG signal generated by the numerical model in the young and healthy reference case and two types of experimental records: using a portable BCG device (x, y, and z) attached to the lumbar region of a subject and using an ultra-low frequency BCG table (x and y) (van der Linde and Knoop, 1966). Except for the absence of a first upward H wave on the artificial signal, the agreement between these three BCG signals on the y axis is very good, with a similar amplitude and position of the different waves. We also find a relatively similar amplitude on the x axis of the three signals, but it is much more subject to noise, even though some similar patterns are found between the numerical model and the ultra-low frequency table. Finally, on the z axis, while there is no ultra-low frequency BCG table data available for comparison, the portable BCG signal seems to be highly impacted by a phenomenon that is not considered in the numerical model. We assume that the large waves observed on the z axis of the portable BCG signal correspond either to a contamination by the seismocardiographic signal or, most probably, by the propagation of a pulse wave through a vessel located just below the BCG sensor. It is thus very likely that this discrepancy between the numerical model and the portable BCG records are the consequence of some particularities of the portable BCG technique rather than a misestimation of the movement of the center of mass induced by cardiovascular activity.

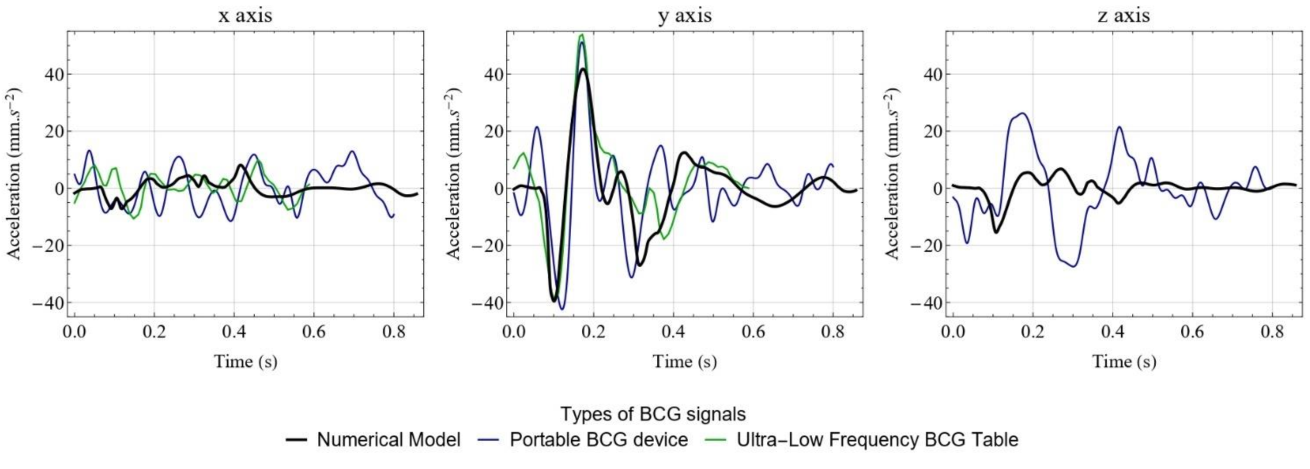

Supplementary Figure 1. Comparison of the 3D acceleration BCG signal given by the numerical model with typical experimental signals given by a portable BCG device (x, y, and z) attached to the lumbar region of a subject and by an ultra-low frequency BCG table (x and y). Data for the ultra-low frequency BCG table are taken from (van der Linde and Knoop, 1966).

6 Complementary results related to pulse wave velocity

6.1 Determination of the scaling law

Based on Equations 15 and A4, and the fact that the I, J, and K peaks are mainly caused by the propagation of blood flow along the aorta, we can write that in the aorta:

$$\frac{A_{0,\mathrm{Ao}}}{{c_{0,\mathrm{Ao}}}^2}\frac{\partial P_{\mathrm{elast}}(s,t)}{\partial t} \propto -\frac{\partial Q(s,t)}{\partial s} \tag{A12}$$

with $c_{0,\mathrm{Ao}}$ the average pulse wave velocity along the aorta and $A_{0,\mathrm{Ao}}$ the average cross-sectional area at pressure P_{ref} along the aorta.

Between two peaks of the longitudinal $\boldsymbol{BCG}_{\mathbf{acc}}$ signal, separated by a time interval Δt, we assume that the slope of the pressure wave is proportional to $\frac{1}{T_{vcp}}$, and thus to $\frac{1}{\sqrt{T_{RR}}}$. We also assume that the difference in the amplitude of blood flow between two sections of the aorta is proportional to $\frac{1}{LVET}$. Therefore, we can write:

$$\frac{1}{c_{0,Ao}} = \kappa_{PTT}\sqrt{\frac{\Delta t\,\sqrt{T_{RR}}}{A_{0,Ao}\,LVET}} = \kappa_{PTT}\,\widetilde{PTT}_{\Delta t} \tag{A13}$$

with $\widetilde{PTT}_{\Delta t}$ measured in $s^{1/4}.cm^{-1}$ and being equivalent to a pulse transit time after a transformation operated on the Δt time interval between two peaks on the BCG signal, while κ_{PTT} is a proportionality factor measured in $s^{3/4}$.

6.2 Effect of the scaling law

The dataset described in the main text is used to assess the performance of the suggested scaling law (Equation 33) to evaluate the inverse of the mean pulse wave velocity in the aorta. Supplementary Figure 2 presents the results of the correlation analysis based on the time intervals directly measured on the longitudinal axis of $\boldsymbol{BCG}_{\mathbf{acc}}$ after low-pass filtering with a cutoff frequency at 25 Hz. The results based on the same time intervals, but transformed with the suggested scaling law, are already presented in the main text (Figure 9).

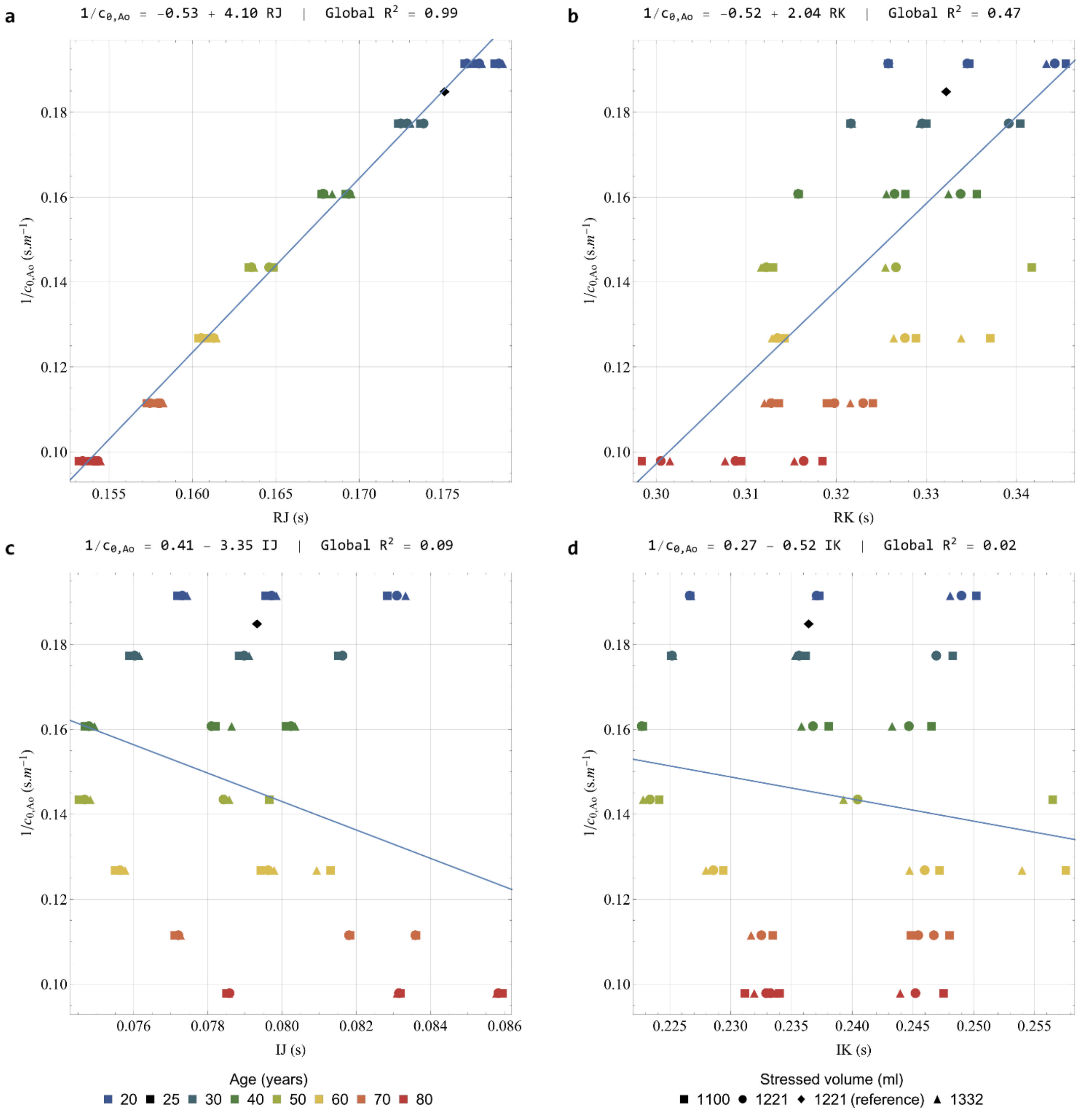

Supplementary Figure 2. Correlation of the inverse of pulse wave velocity in the aorta with a selection of time intervals: a. RJ; b. RK; c. IJ; and d. IK. All the results are obtained after low-pass filtering of the acceleration signal at 25 Hz. In each case, the global linear regression line is indicated together with its equation and the regression coefficient R^2. Colors indicate different simulated ages, while symbols indicate different stressed volumes. Several points can have the same color and symbol, when

they correspond to different heart rates. The healthy young reference case is indicated by a black diamond.

Most of the time intervals presented in Supplementary Figure 2 display very bad correlation coefficients with the inverse of the pulse wave velocity in the aorta. These results differ a lot from those presented in Figure 9, for which the scaling law is used (e.g., $R^2 = 0.02$ for IK vs. $R^2 = 0.98$ for $\widetilde{PTT_{IK}}$). The only exception is the RJ time interval, since its correlation coefficient is already very high without the scaling law ($R^2 = 0.99$). Similar results are also observed for the RI time interval (not presented).

These results overall support the interest of using the scaling law to evaluate pulse wave velocity in the aorta.

6.3 Effect of filtering

It is reasonable to assume that different body types will have different dampening effects on the BCG signal. The sensitivity of a metric to these dampening effects can be studied by looking at different filters applied to the initial simulated BCG signals. Here we consider two low-pass filters with a cutoff frequency of 25 and 40 Hz, respectively. Figure 6 already shows the impact that these filters have on $\boldsymbol{BCG}_{\mathbf{vel}}$ and $\boldsymbol{BCG}_{\mathbf{acc}}$.

It has already been shown that the different time intervals measured on $\boldsymbol{BCG}_{\mathbf{acc}}$ were only slightly affected by low-pass filters, down to a cutoff frequency of 25 Hz (Gómez-Clapers et al., 2013). Supplementary Figure 3 presents the Bland-Altman plots used to compare the results obtained with the scaling law, based on a low-pass filtered $\boldsymbol{BCG}_{\mathbf{acc}}$ signal with a cutoff frequency at 40 versus 25 Hz. The mean differences observed on all the parameters studied are very close to 0, even though $\widetilde{PTT_{IJ}}$ appears to be a bit overestimated with a cutoff frequency at 25 Hz, in comparison to 40 Hz. Even for this parameter, however, the mean difference is close to 10% of the mean value observed for $\widetilde{PTT_{IJ}}$ in all the simulated cases. Such a difference can be considered relatively low in comparison with the wide range of values taken for the inverse of pulse wave velocity in the aorta (twice as large at 20 years old as at 80 years old). This confirms the potential of this scaling law to provide parameters that are only slightly affected by different body types and can allow the estimation of some vascular parameters such as pulse wave velocity in the aorta.

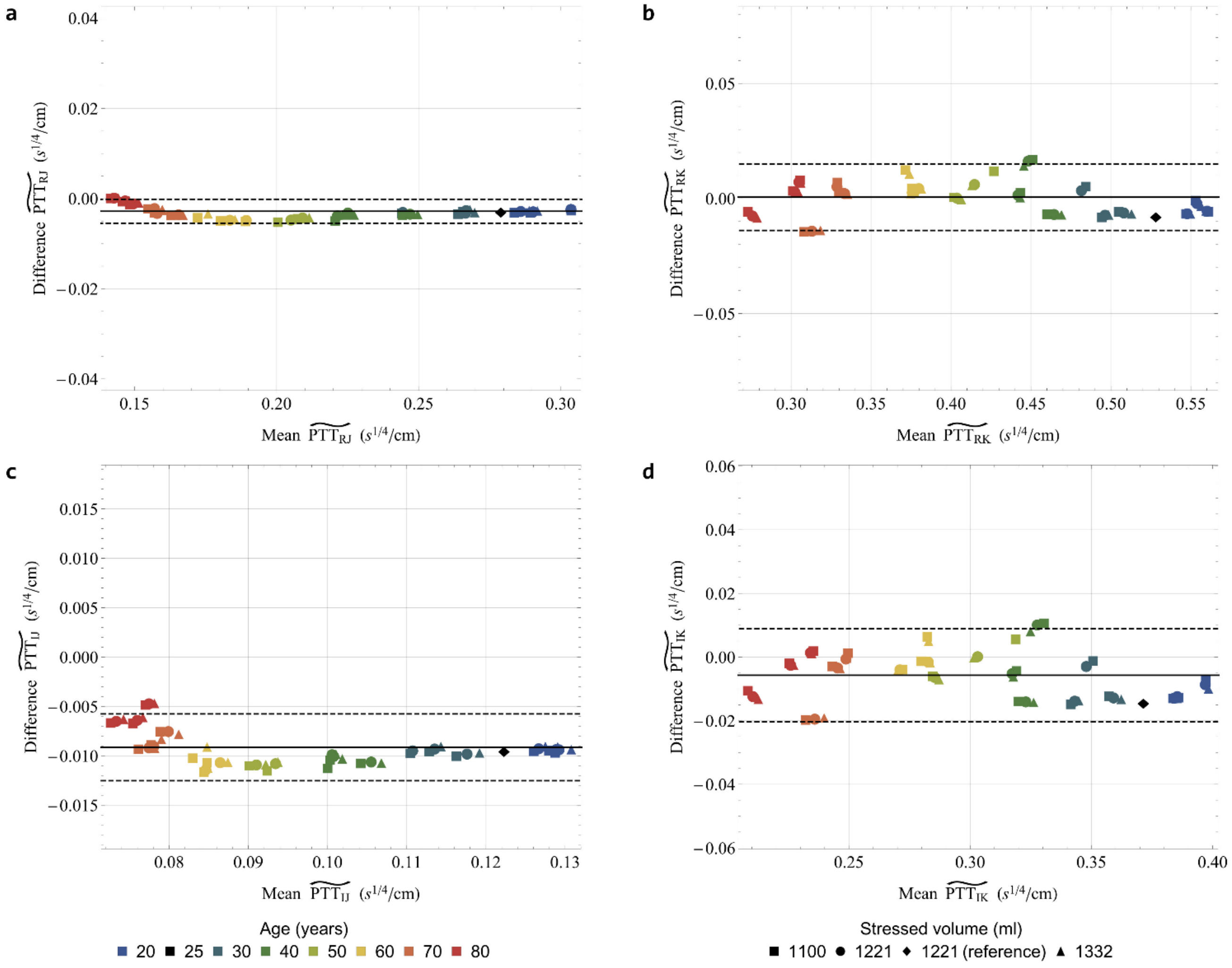

Supplementary Figure 3. Bland-Altman plots comparing results obtained based on a low-pass filtered $\boldsymbol{BCG}_{\mathbf{acc}}$ signal with a cutoff frequency at 40 and 25 Hz for: a. $\widetilde{PTT}_{RJ}$; b. $\widetilde{PTT}_{RK}$; c. $\widetilde{PTT}_{IJ}$; and d. $\widetilde{PTT}_{IK}$. A positive difference indicates that the parameter of interest is larger with the cutoff frequency at 40 Hz than the one at 25 Hz. The mean difference is indicated by a solid line, while the confidence interval (mean +/- 1.96 standard deviations) is indicated with dashed lines. The range on the vertical axis corresponds to +/-20% of the mean value observed for a given parameter. Colors indicate different simulated ages, while symbols indicate different stressed volumes. Several points can have the same color and symbol, when they correspond to different heart rates. The healthy young reference case is indicated by a black diamond.

7 Complementary results related to stroke volume

7.1 Determination of a scaling law linked to the integral of the kinetic energy

The scaling law giving SV_1 (Equation 34) has a physical basis described in the literature (Starr et al., 1939), and the one giving SV_2 (Equation 35) is an adaptation that may give more robust results, based on some in silico observations, as described in Section 3.4.2. The scaling law giving SV_4 (Equation 37) corresponds to another modification of Equation 34, based on the fact that iK_{sys} should be less sensitive

to inter-individual differences than the area under the I and J waves. However, it does not have a direct physical basis, as opposed to the scaling law giving SV_3 (Equation 36), which relies on the development of some relationships that are described hereafter.

Since the aorta is the largest contributor to $\boldsymbol{BCG}_{\mathbf{vel}}$, and thus BCG_{kin}, the following reasoning can be conducted based on Equation 22:

$$\|\boldsymbol{BCG}_{\mathbf{vel}}(t)\| \propto \frac{\rho\, SV}{W_b} \frac{Q_{AV}(t)}{A_{0,\mathrm{Ao}}} \tag{A14}$$

with Q_{AV} the blood flow rate through the aortic valve.

This means that:

$$BCG_{\mathrm{kin}} \propto \frac{\rho^2\, SV^2}{2\, W_b} \frac{Q_{AV}(t)^2}{A_{0,\mathrm{Ao}}^2} \tag{A15}$$

Furthermore, we can make the usual approximation that Q_{AV} has the shape of a squared sinus of amplitude Q_{max} and width $LVET$. By integrating Equation A15 over the systolic phase (i.e., for BCG_{kin}, from the beginning of the cardiac cycle until the time corresponding to the end of the 'J wave), we find:

$$iK_{\mathrm{sys}} \propto \frac{\rho^2\, SV^2}{2\, W_b\, A_{0,\mathrm{Ao}}^2} \int_0^{LVET} Q_{\mathrm{max}}^2 \sin\left(\frac{\pi\, t}{LVET}\right)^4 dt \tag{A16}$$

Since the stroke volume is also defined as the integral of Q_{AV} over the ejection phase, we can find that:

$$Q_{\mathrm{max}} = \frac{2\, SV}{LVET} \tag{A17}$$

Injecting this expression in Equation A16 and solving the integral, we get:

$$iK_{\mathrm{sys}} \propto \frac{3\, \rho^2\, SV^4}{4\, W_b\, A_{0,Ao}^2\, LVET} \tag{A18}$$

which leads to the scaling law given in Equation 36.

7.2 Effect of filtering on the results related to stroke volume

The metrics based on the amplitude of the BCG signals are also susceptible to be affected by filtering, and thus, as hypothesized, different body types. In this section, we are particularly interested about the estimates of stroke volumes obtained from $\boldsymbol{BCG}_{\mathbf{acc}}$ and $\boldsymbol{BCG}_{\mathbf{kin}}$, according to the equations proposed in Section 3.4.2.

Supplementary Figure 4 presents the Bland-Altman plots used to compare the stroke volume estimates, based on an initial low-pass filtered $\boldsymbol{BCG}_{\mathbf{acc}}$ signal with a cutoff frequency at 40 versus 25 Hz. The mean differences observed on all the stroke volume estimates are very small (below 10% of the mean value observed for all the simulated cases) and always positive. This indicates that the stroke volumes computed with a cutoff frequency at 25 Hz are underestimated, in comparison to 40 Hz. The scatter around the mean difference is very small for the four different formulas of stroke volume, but SV_3 appears to perform better, with a mean difference very close to 0. More generally, estimates based on $\boldsymbol{BCG}_{\mathbf{kin}}$ seem to be less affected by filtering than those based on $\boldsymbol{BCG}_{\mathbf{acc}}$. This was to be expected, because the $\boldsymbol{BCG}_{\mathbf{acc}}$ signal contains higher frequencies and the integration that leads to $\boldsymbol{BCG}_{\mathbf{vel}}$ (and thus $\boldsymbol{BCG}_{\mathbf{kin}}$) already plays a role of low-pass filter.

This short analysis confirms the potential of the suggested formulas to evaluate stroke volume and its changes with only a low impact of different body types on the estimates. Since the bias induced by low-pass filtering appears to be very stable, one could imagine that empirical laws could provide a correcting factor to apply to the BCG estimates, based on physical parameters of the subject.

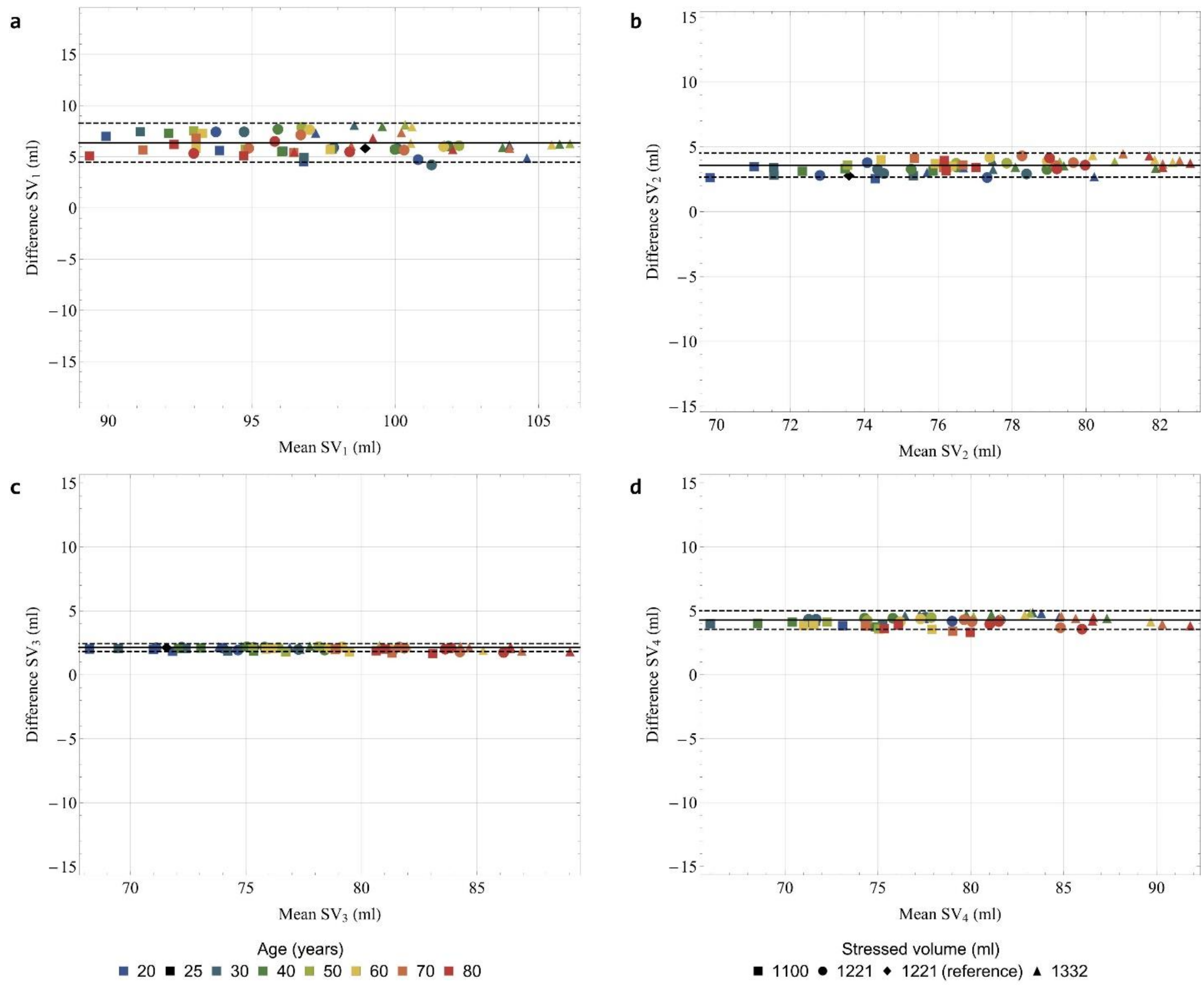

Supplementary Figure 4. Bland-Altman plots comparing results obtained based on a low-pass filtered $\boldsymbol{BCG}_{\mathbf{acc}}$ signal with a cutoff frequency at 40 and 25 Hz for: a. SV_1 (Equation 34); b. SV_2 (Equation 35); c. SV_3 (Equation 36); and d. SV_4 (Equation 37). A positive difference indicates that the parameter of interest is larger with the cutoff frequency at 40 Hz than the one at 25 Hz. The mean difference is indicated by a solid line, while the confidence interval (mean +/- 1.96 standard deviations) is indicated with dashed lines. The range on the vertical axis corresponds to +/-20% of the mean value observed for a given parameter. Colors indicate different simulated ages, while symbols indicate different stressed volumes. Several points can have the same color and symbol, when they correspond to different heart rates. The healthy young reference case is indicated by a black diamond.

7.3 Effect of peripheral resistance on the estimation of stroke volume

Since peripheral resistance has an influence on stroke volume, we evaluate its impact on the quality of the estimates provided by the four suggested formulas. This analysis is conducted on the young healthy reference case, by gradually increasing the value of each systemic peripheral resistance (computed as

$R_0 + R_1$) up to three times its initial value. The corresponding results are provided in Supplementary Figure 5.

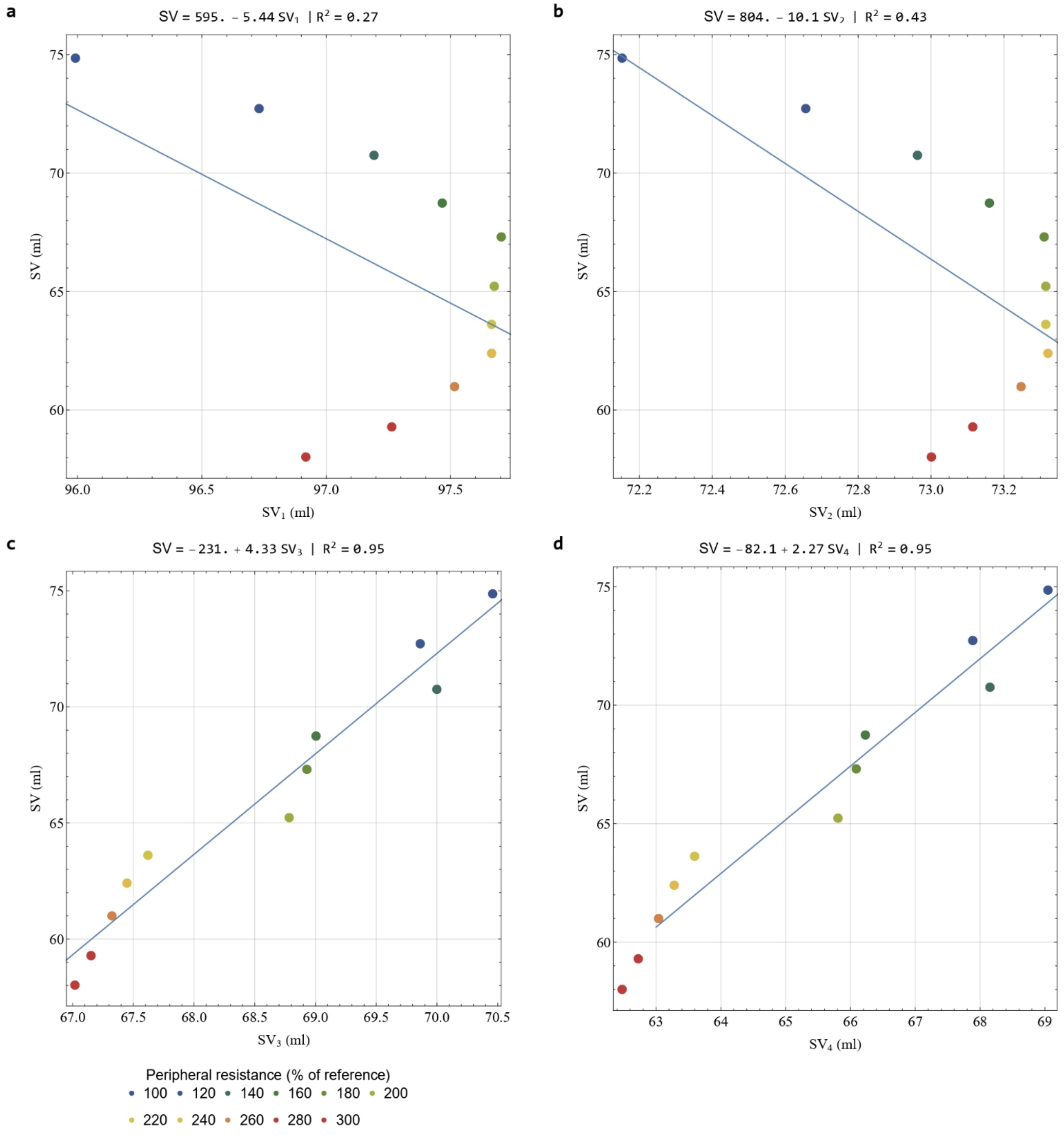

Supplementary Figure 5. Correlation of actual stroke volume with estimated stroke volumes obtained after changing the total systemic peripheral resistance: a. $\boldsymbol{SV_1}$ (Equation 34); b. $\boldsymbol{SV_2}$ (Equation 35); c.

$\boldsymbol{SV_3}$ (Equation 36); and d. $\boldsymbol{SV_4}$ (Equation 37). All the results are obtained after low-pass filtering of the acceleration signal at 25 Hz. R^2: correlation coefficient computed on the whole dataset

While SV_1 and SV_2 are visibly not able to correctly evaluate intra-individual changes of stroke volume caused by an increase in systemic peripheral resistance, SV_3 and SV_4 seem to keep a linear relationship with regard to stroke volume ($R^2 = 0.95$). However, the estimated changes are relatively underestimated in both cases (slope of 4.33 for SV_3 and 2.27 for SV_4).

References

Alastruey, J., Parker, K. H., and Sherwin, S. J. (2012). “Arterial pulse wave haemodynamics,” in *11th International Conference on Pressure Surges*, ed. S. Anderson (Bedford, UK: British Hydromechanics Research (BHR) Group), 401–443.

Baan, J., Cunningham, D. M., and Noordergraaf, A. (1966). “The Meaning of the Human Lateral Ballistocardiogram,” in *Ballistocardiography and Cardiovascular Dynamics* (Amsterdam), 274–280.

Bogren, H. G., and Buonocore, M. H. (1999). 4D magnetic resonance velocity mapping of blood flow patterns in the aorta in young vs. elderly normal subjects. *J. Magn. Reson. Imaging* 10, 861–869. doi:10.1002/(SICI)1522-2586(199911)10:5<861::AID-JMRI35>3.0.CO;2-E.

Buess, A., Van Muylem, A., Nonclercq, A., and Haut, B. (2020). Modeling of the Transport and Exchange of a Gas Species in Lungs With an Asymmetric Branching Pattern. Application to Nitric Oxide. *Front. Physiol.* 11. doi:10.3389/fphys.2020.570015.

Gallo, C., Ridolfi, L., and Scarsoglio, S. (2020). A Closed-Loop Multiscale Model of the Cardiovascular System: Application to Heart Pacing and Open-Loop Response. in *XV Mediterranean Conference on Medical and Biological Engineering and Computing – MEDICON 2019* IFMBE Proceedings., eds. J. Henriques, N. Neves, and P. de Carvalho (Cham: Springer International Publishing), 577–585. doi:10.1007/978-3-030-31635-8_69.

Gómez-Clapers, J., Serra Rocamora, A., Casanella Alonso, R., and Pallàs Areny, R. (2013). Uncertainty factors in time-interval measurements in ballistocardiography. in, 395–399. Available at: https://upcommons.upc.edu/handle/2117/21545 [Accessed March 30, 2021].

Liang, F., Takagi, S., Himeno, R., and Liu, H. (2009). Multi-scale modeling of the human cardiovascular system with applications to aortic valvular and arterial stenoses. *Med. Biol. Eng. Comput.* 47, 743–755. doi:10.1007/s11517-009-0449-9.

Mynard, J. P., and Smolich, J. J. (2015). One-Dimensional Haemodynamic Modeling and Wave Dynamics in the Entire Adult Circulation. *Ann. Biomed. Eng.* 43, 1443–1460. doi:10.1007/s10439-015-1313-8.

Pedley, T. J., Schroter, R. C., and Sudlow, M. F. (1970a). Energy losses and pressure drop in models of human airways. *Respir. Physiol.* 9, 371–386. doi:10.1016/0034-5687(70)90093-9.

Pedley, T. J., Schroter, R. C., and Sudlow, M. F. (1970b). The prediction of pressure drop and variation of resistance within the human bronchial airways. *Respir. Physiol.* 9, 387–405. doi:10.1016/0034-5687(70)90094-0.

Seed, W. A., and Wood, N. B. (1971). Velocity patterns in the aorta. *Cardiovasc. Res.* 5, 319–330. doi:10.1093/cvr/5.3.319.

Starr, I., Rawson, A. J., Schroeder, H. A., and Joseph, N. R. (1939). Studies on the estimation of cardiac ouptut in man, and of abnormalities in cardiac function, from the heart's recoil and the blood's impacts; the ballistocardiogram. *Am. J. Physiol.-Leg. Content* 127, 1–28. doi:10.1152/ajplegacy.1939.127.1.1.

Tortoli, P., Bambi, G., Guidi, F., and Muchada, R. (2002). Toward a better quantitative measurement of aortic flow. *Ultrasound Med. Biol.* 28, 249–257. doi:10.1016/S0301-5629(01)00462-8.

van der Linde, J., and Knoop, A. A. (1966). "Significance of the Frontal Vector Ultra-Low Frequency Acceleration Ballistocardiogram of Man," in *Ballistocardiography and Cardiovascular Dynamics* (Amsterdam), 78–84.
